# Supplementary material for: Dissecting autonomous enzymatic variability in single cells
Source: Nat Commun. 2026 Jul 2;17:5788. doi: 10.1038/s41467-026-74172-z (PMC13328634; doi:10.1038/s41467-026-74172-z)
Supplement: Supplementary file 1 — Supplementary Information [file 41467_2026_74172_MOESM1_ESM.pdf]

Supplementary Information

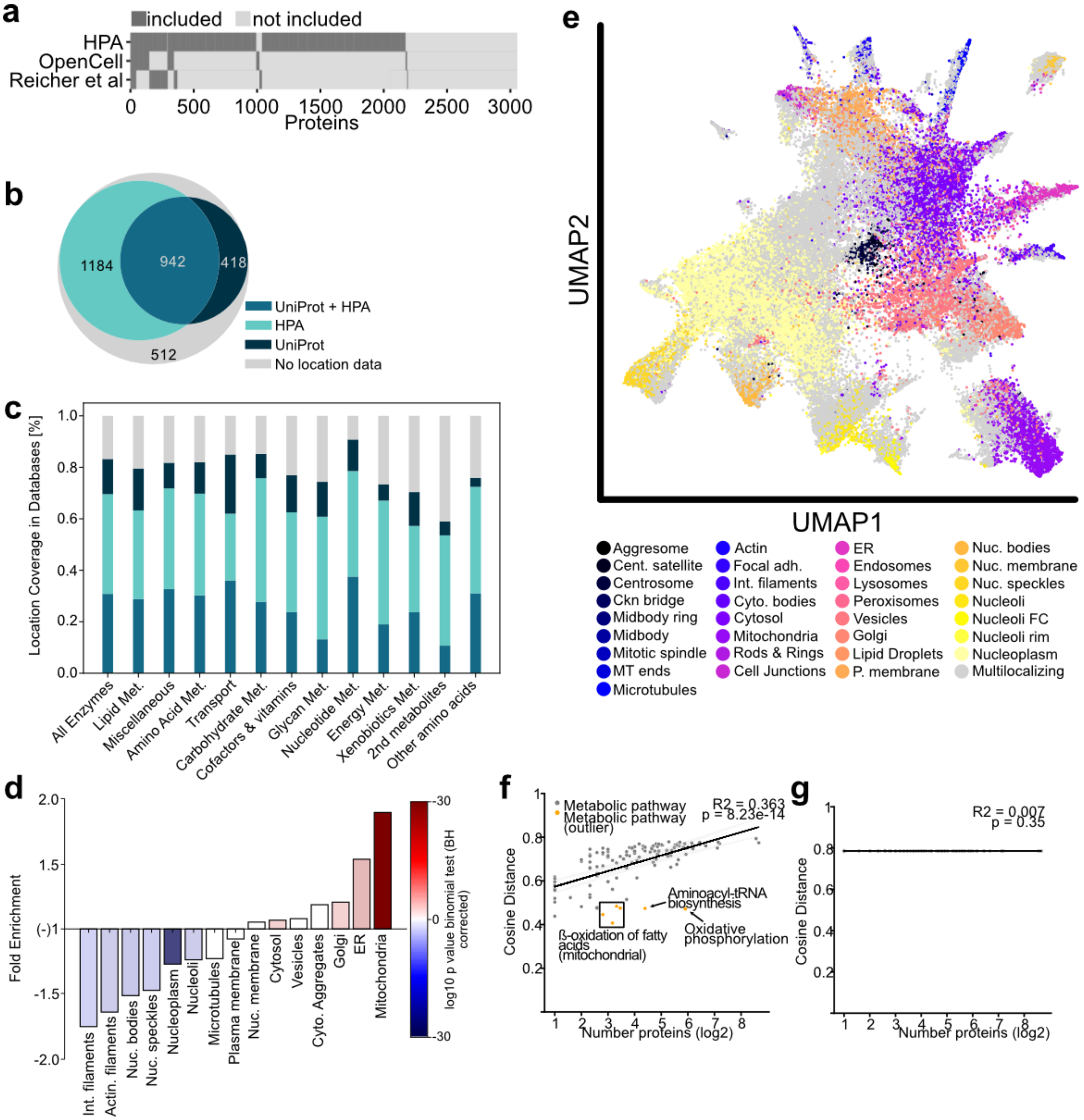

Fig S1: The metabolic proteome in the HPA and in UniProt

a, Coverage of metabolic enzymes across pathway groups in the HPA Subcellular Section, OpenCell[16] and Reicher et al[27]. Source data are provided as a Source Data file. **b,** Coverage of metabolic enzymes in the HPA subcellular section and UniProt. **c,** Coverage of metabolic enzymes across pathway groups in the HPA subcellular section and UniProt. Source data are provided as a Source Data file. **d,** Overrepresentation analysis for enzyme localisation in the HPA dataset compared to the background of the human proteome. Enrichment was assessed using two-sided binomial tests and resulting p-values were adjusted for multiple comparisons using the Benjamini–Hochberg procedure. Source data are provided as

a Source Data file. **e,** UMAP visualization of the image features of the entire HPA image dataset from Ouyang, *et al.*[30] All proteins are displayed. Single location images are colored according to location, while gray data points belong to multilocalizing proteins. **f,** Logarithmic correlation between the cosine distances from Fig. 1d and the number of proteins in a pathway. The regression line was calculated using ordinary least squares (OLS) on the log-transformed number of proteins. Standardized residuals were used to identify potential outliers, defined as pathways with residuals exceeding 2 standard deviations from the mean; these pathways are highlighted in the plot. Source data are provided as a Source Data file. **g,** Scatterplot of random cosine distances from Fig. 1d and the number of proteins in a pathway reveals no correlation. Source data are provided as a Source Data file.

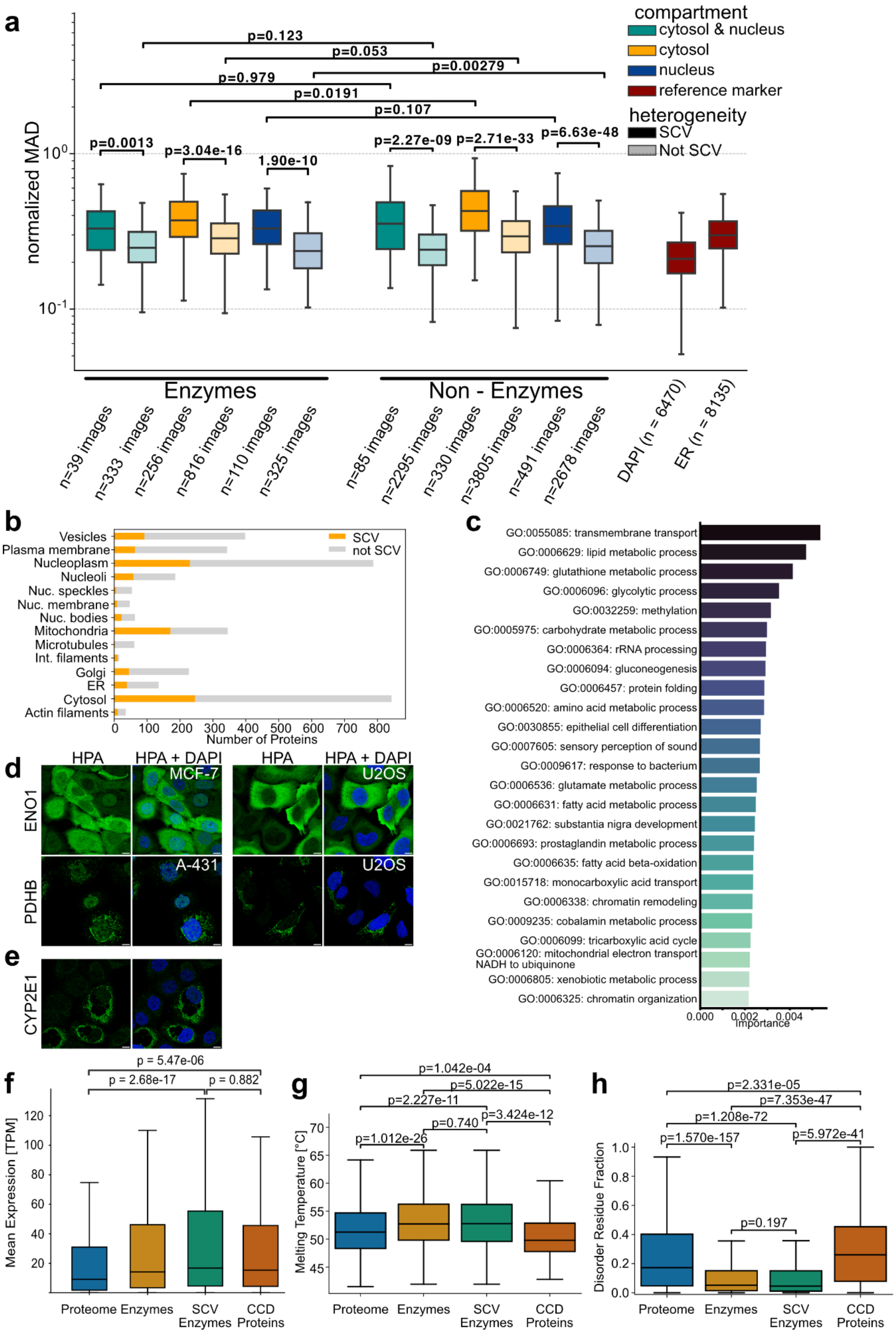
a
compartment
cytosol & nucleus
cytosol
nucleus
reference marker
heterogeneity
SCV
Not SCV
normalized MAD
10^0
10^{-1}
p=0.123
p=0.053
p=0.00279
p=0.979
p=0.0191
p=0.107
p=0.0013
p=3.04e-16
1.90e-10
p=2.27e-09
p=2.71e-33
p=6.63e-48
Enzymes
Non - Enzymes
n=39 images
n=333 images
n=256 images
n=816 images
n=110 images
n=325 images
n=85 images
n=2295 images
n=330 images
n=3805 images
n=491 images
n=2678 images
DAPI (n = 6470)
ER (n = 8135)
b
Vesicles
Plasma membrane
Nucleoplasm
Nucleoli
Nuc. speckles
Nuc. membrane
Nuc. bodies
Mitochondria
Microtubules
Int. filaments
Golgi
ER
Cytosol
Actin filaments
SCV
not SCV
0 100 200 300 400 500 600 700 800
Number of Proteins
c
GO:0055085: transmembrane transport
GO:0006629: lipid metabolic process
GO:0006749: glutathione metabolic process
GO:0006096: glycolytic process
GO:0032259: methylation
GO:0005975: carbohydrate metabolic process
GO:0006364: rRNA processing
GO:0006094: gluconeogenesis
GO:0006457: protein folding
GO:0006520: amino acid metabolic process
GO:0030855: epithelial cell differentiation
GO:0007605: sensory perception of sound
GO:0009617: response to bacterium
GO:0006536: glutamate metabolic process
GO:0006631: fatty acid metabolic process
GO:0021762: substantia nigra development
GO:0006693: prostaglandin metabolic process
GO:0006635: fatty acid beta-oxidation
GO:0015718: monocarboxylic acid transport
GO:0006338: chromatin remodeling
GO:0009235: cobalamin metabolic process
GO:0006099: tricarboxylic acid cycle
GO:0006120: mitochondrial electron transport NADH to ubiquinone
GO:0006805: xenobiotic metabolic process
GO:0006325: chromatin organization
0.000 0.002 0.004
Importance
d
HPA
HPA + DAPI
HPA
HPA + DAPI
MCF-7
U2OS
A-431
U2OS
ENO1
PDHB
e
CYP2E1
f
p = 5.47e-06
p = 2.68e-17
p = 0.882
Mean Expression [TPM]
20 40 60 80 100 120
Proteome
Enzymes
SCV Enzymes
CCD Proteins
g
p=1.042e-04
p=5.022e-15
p=2.227e-11
p=0.740
p=3.424e-12
p=1.012e-26
Melting Temperature [°C]
45 50 55 60 65 70
Proteome
Enzymes
SCV Enzymes
CCD Proteins
h
p=2.331e-05
p=7.353e-47
p=1.208e-72
p=1.570e-157
p=5.972e-41
p=0.197
Disorder Residue Fraction
0.0 0.2 0.4 0.6 0.8 1.0
Proteome
Enzymes
SCV Enzymes
CCD Proteins

Fig S2: The variable metabolic proteome in the HPA Subcellular Section

a, The normalized median absolute deviation was calculated between individual cells in the same image as a measure of protein expression variability for different groups of proteins (enzymes and proteins in different cellular compartment that are variably expressed or not based on manual annotations from HPAv23. Source data and detailed statistics included number of samples per group, Benjamini Hochberg-adjusted p values and cliff's delta effect sizes are provided as a Source Data file. **b,** Degree of variability across cellular localizations for the metabolic proteome. Source data are provided as a Source Data file. **c**, The predictive power of GO terms (Biological Process) is low for the prediction of protein expression variability. However, the predictive power was limited (F1 score = 0.353). Top 100 terms are provided in the source data file. **d,** Immunofluorescence images for ENO1 (top) and PDHB (bottom) reveal conserved single-cell heterogeneity across multiple cell lines. **e,** Immunofluorescence images of CYP2E1 reveal single-cell heterogeneity. **f-h,** Enzymes have different physical properties from CCD proteins. **f,** Heterogeneously expressed enzymes and CCD proteins have comparable RNA expression levels. **g, h** Differences in physical properties, indicated by protein melting points **(g)** and protein disorder **(h)** between variable enzymes and cell cycle dependent proteins. Mann-Whitney U test with Benjamini-Hochberg correction. For box plots: center line, median; box, first (Q1) and third (Q3) quartiles; whiskers, 1.5× interquartile range (IQR) below Q1 and above Q3. Number of datapoints Proteome/Enzymes/SCV Enzymes/CCD Proteins: **(f)** 10063/1747/677/275; **(g)** 7513/1559/630/205; **(h)** 12793/2116/803/314 . Raw data underlying these figures as well as Benjamini-Hochberg adjusted p values and cliffs delta effect sizes are provided in the source data file.

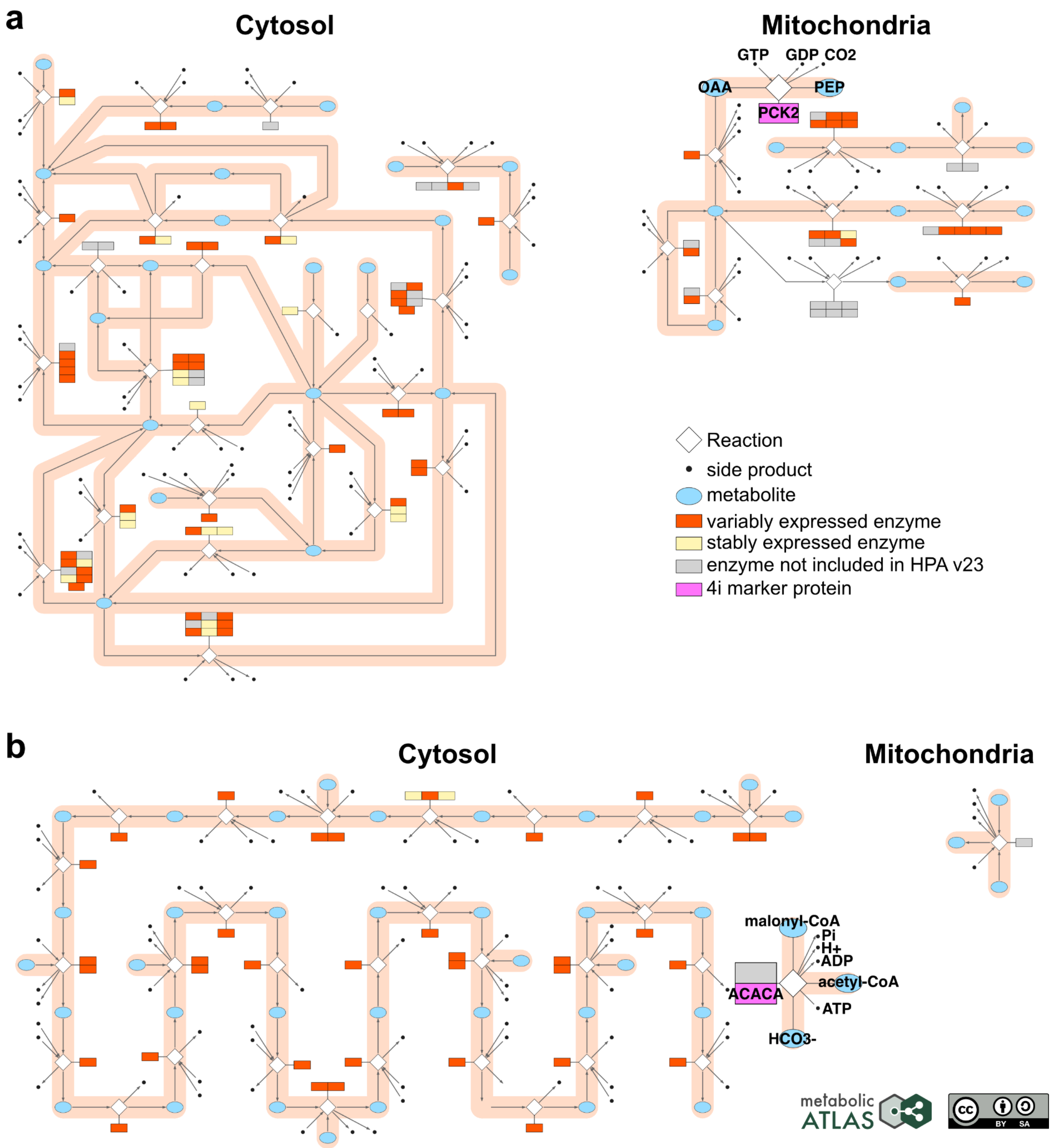

Fig S3: PCK2 and ACACA are variably expressed, rate-limiting enzymes.
Human1 pathway diagrams obtained from the Metabolic Atlas for **a**, pyruvate metabolism (rate limiting enzyme PCK2) and **b**, even chain fatty acid biosynthesis (rate limiting enzyme ACACA) reveal pathway-wide single cell variability of protein expression. Variably expressed enzymes are visualized with red boxes, stably expressed enzymes in yellow boxes, enzymes not included in HPAv23 in grey boxes. Protein expression variability can be explored for all other metabolic pathways using the interactive pathway explorer at https://www.proteinatlas.org/humanproteome/subcellular/metabolic+proteome.

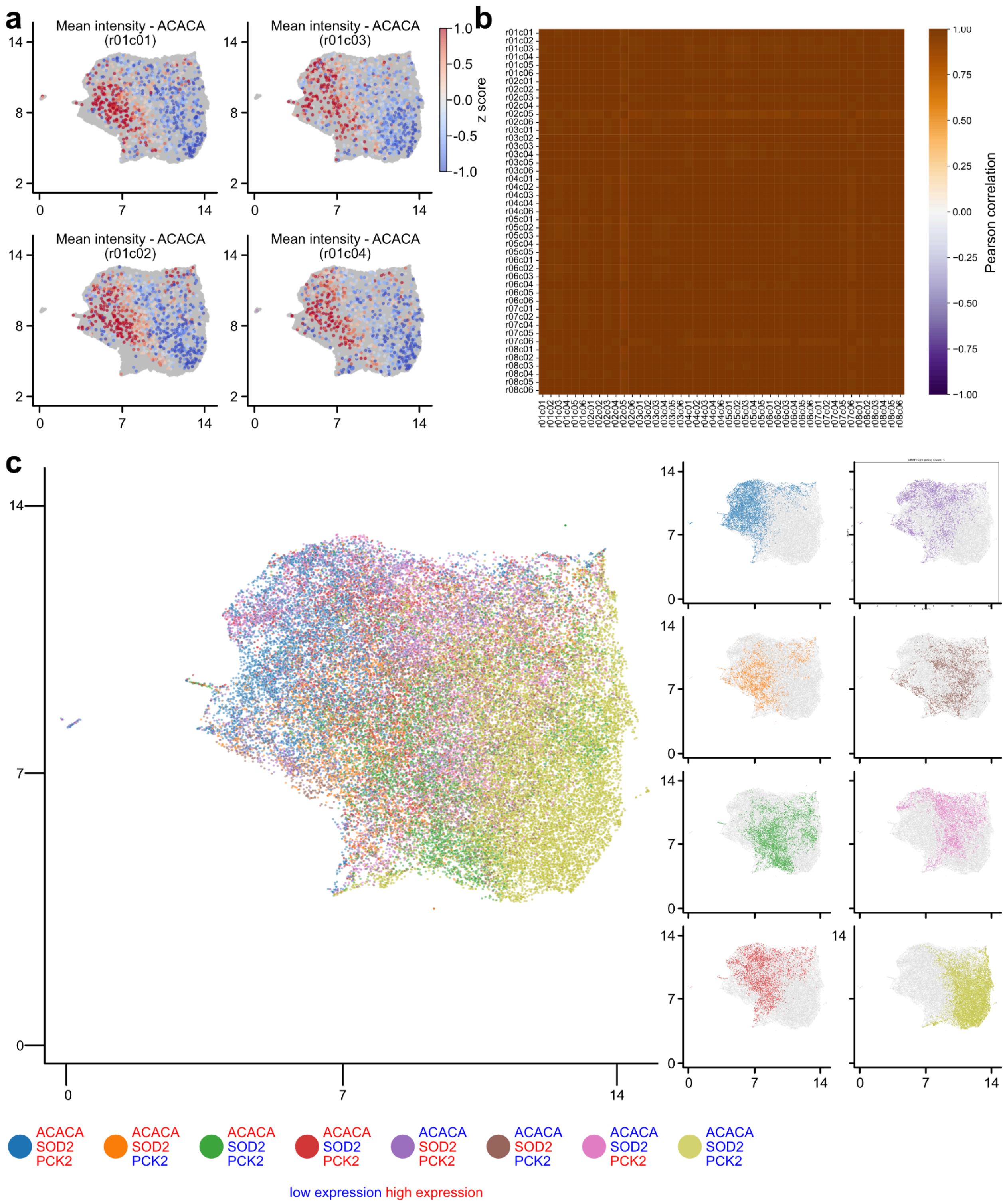

Fig S4: Quality control of 4i experiment.

a, Mean ACACA expression visualized in two dimensional cellular state landscape is highly similar across 4 replicate wells **b**, The median value was calculated for each feature and compared between all replicate wells using Pearson correlation. Average pairwise Pearson correlation >0.99. **c**, Cells were

assigned to 8 different metabolic states based on high or low expression of ACACA, SOD2 and PCK2. Each dot in the UMAP corresponds to a cell and is colored by the resulting metabolic state.

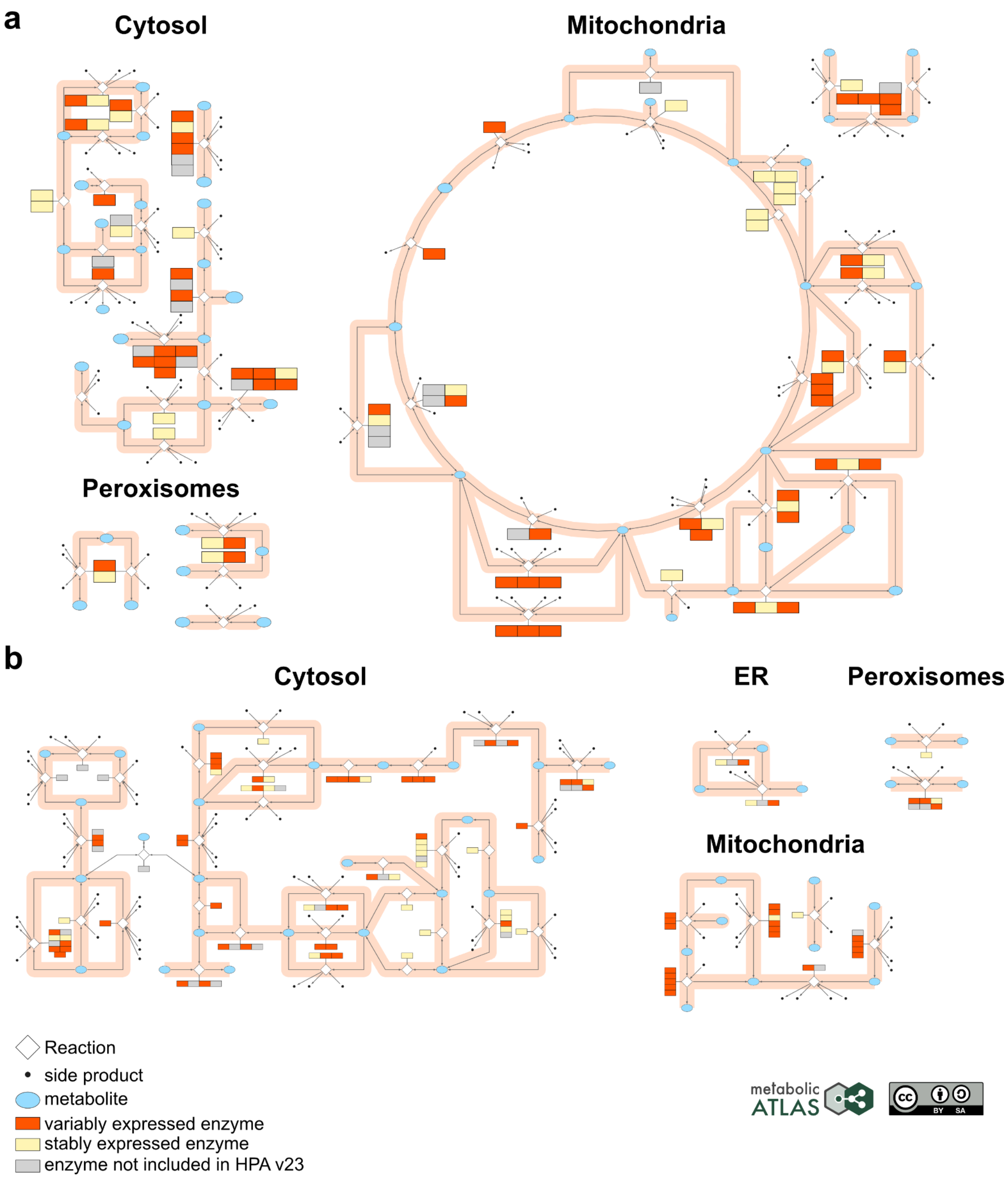

Fig S5: Reach of metabolic variability

Human1 pathway diagrams obtained from the Metabolic Atlas highlight metabolic reactions performed by variably expressed metabolic enzymes in **a**, the TCA cycle **b**, and gluconeogeneisis/glycolysis. Variably expressed enzymes are visualized with red boxes, stably expressed enzymes in yellow boxes, enzymes not included in HPAv23 in grey boxes. Protein expression variability can be explored for all other metabolic pathways using the interactive pathway explorer at https://www.proteinatlas.org/humanproteome/subcellular/metabolic+proteome.

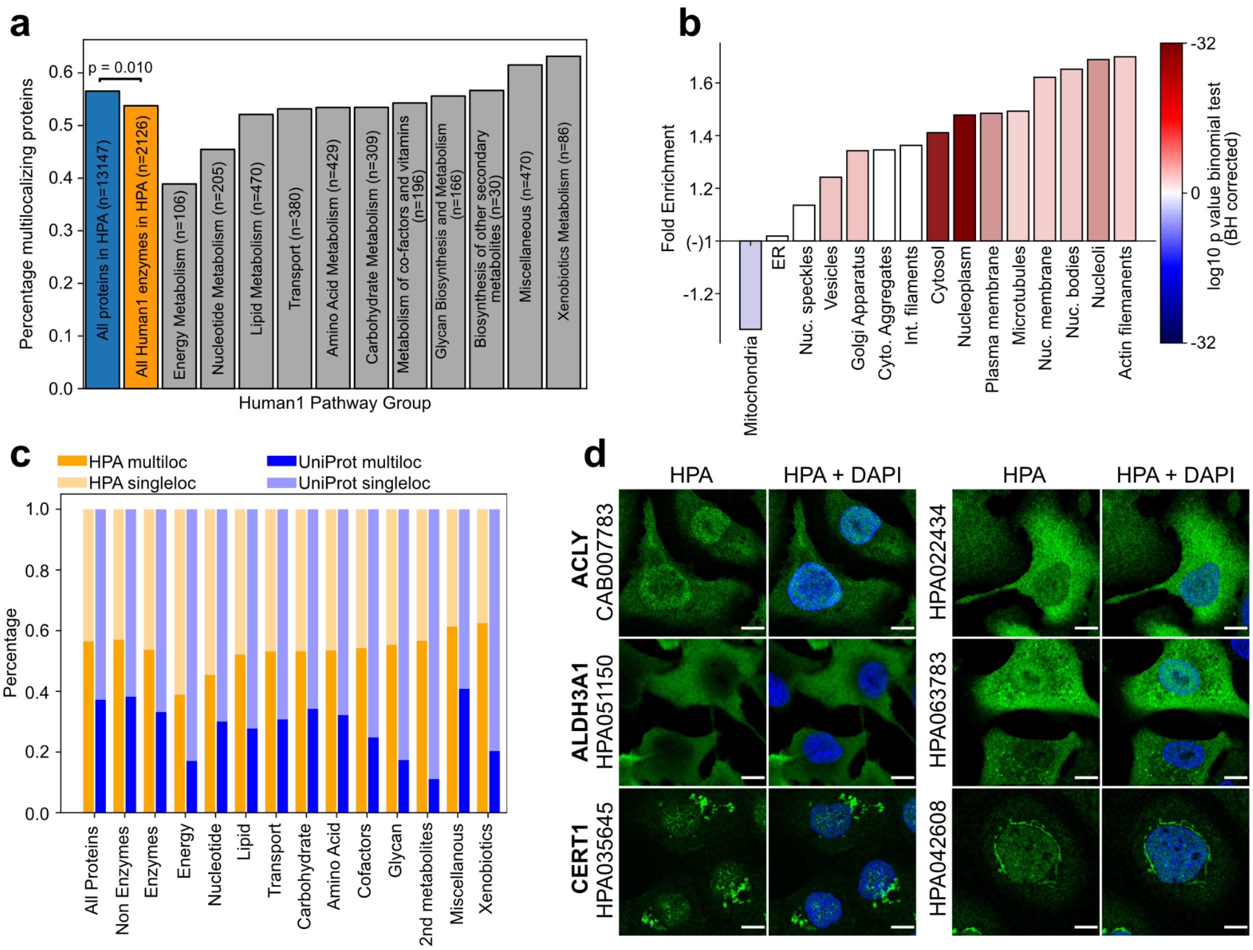

Fig S6: Multilocalizing proteins are understudied.

a, Enzymes exhibit a lower degree of multilocalization compared to the human proteome (two-sided binomial test, p=0.010, n =13147 (All Proteins) and 2126 (Enzymes). **b,** Enrichment was assessed using two-sided binomial tests and resulting p-values were adjusted for multiple comparisons using the Benjamini–Hochberg procedure. Source data are provided as a Source Data file. **c,** The UniProt database contains a lower number of multilocalizing proteins compared to the HPA Subcellular Section. **d,** Example images for enzymes without location data in UniProt that have multiple locations in the HPA. Results were validated with independent antibody stainings; blue = DAPI, green = protein of interest; scale bar corresponds to 10 µm.

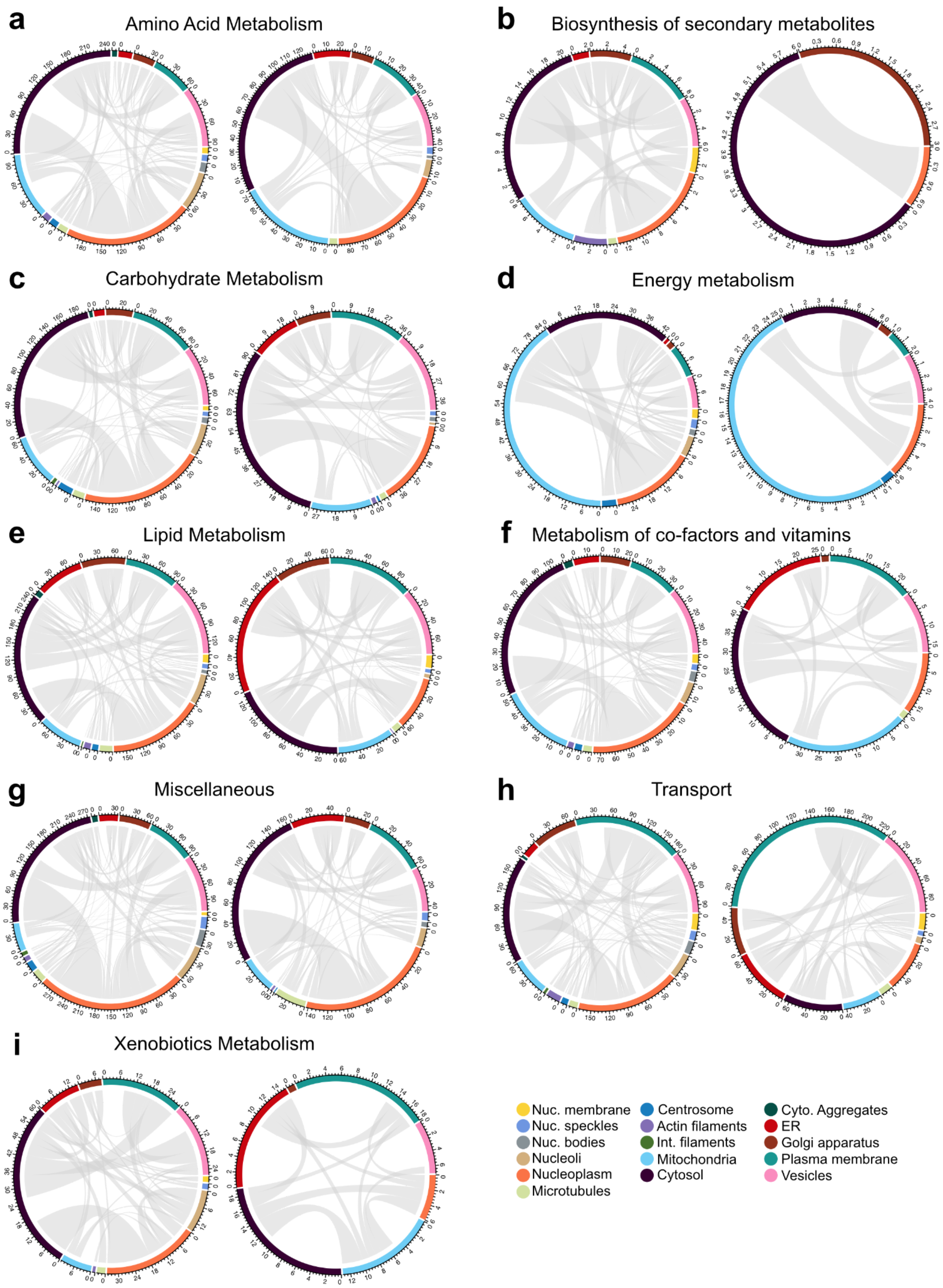

Fig S7: Spatial complexity of the metabolic proteome.

a-i Circos plots displaying subcellular distribution of metabolic proteins per pathway group in the HPA dataset (left plots) and with experimental location evidence on UniProt (right plots). A connection between subcellular locations indicates multilocalization.

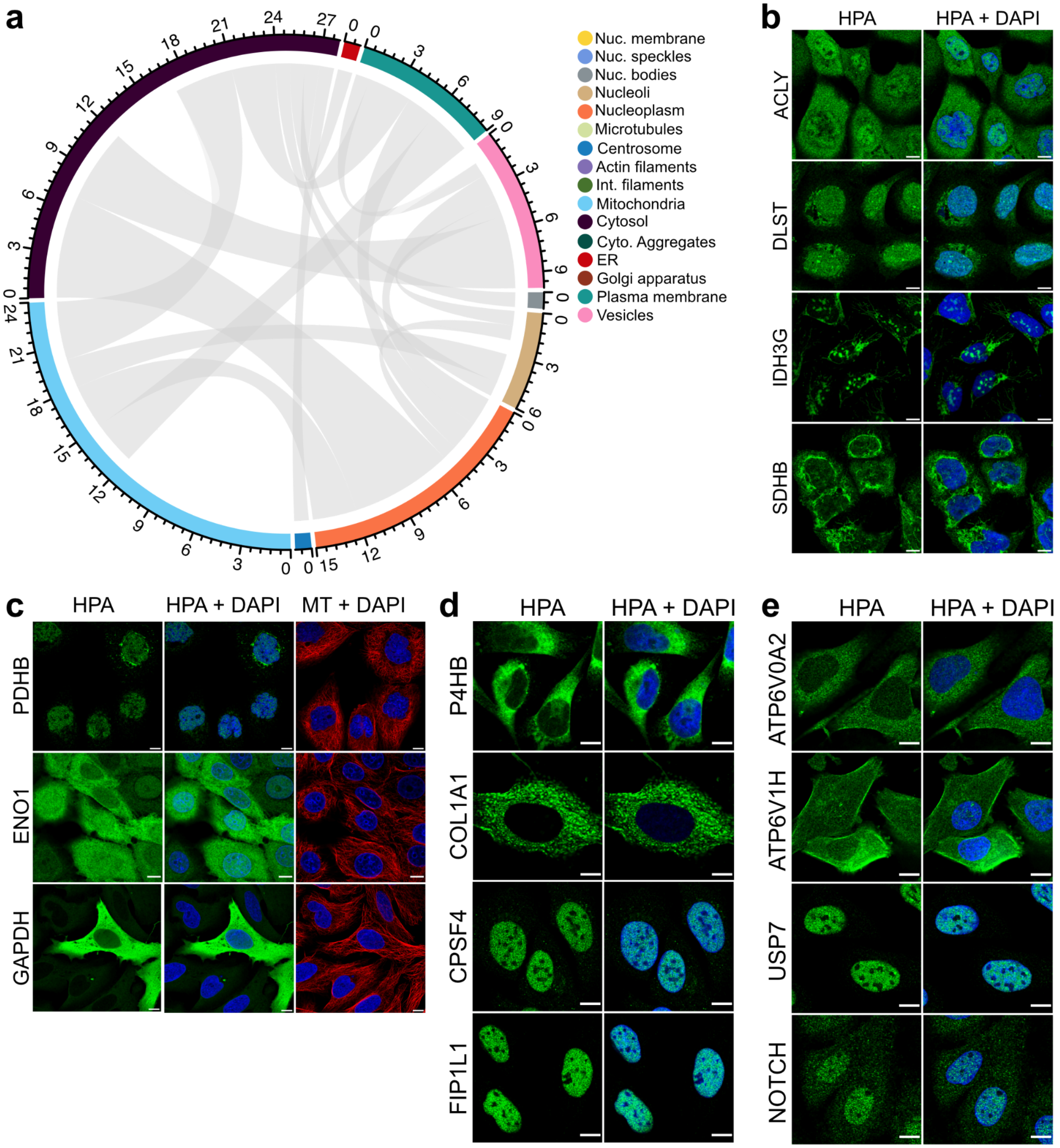

Fig S8: Multifunctional proteins.

a, Circos plots displaying subcellular distribution of metabolic enzymes in the TCA cycle. A connection between subcellular locations indicates multilocalization. **b**) Example images for TCA cycle enzymes with additional nuclear localization (blue = DAPI, green = protein of interest, scale bar corresponds to 10 µm). **c**) Example images of known multifunctional proteins exhibiting single cell heterogeneity (blue = DAPI, green = protein of interest, red = microtubules; scale bar corresponds to 10 µm). **d,** Immunofluorescence images of proteins interacting with P4HA2. P4HB and COL1A1 are cytoplasmic proteins involved in collagen synthesis. CPSF4 and FIP1L1 are members of the nuclear CPSF splicing

complex. **e**, Immunofluorescence images of proteins interacting with ATP6V1D. ATP6V0A2 and ATP6V1H are cytoplasmic proteins, USP7 and NOTCH localize to the nucleus.

Table S1: Collapsed annotations for HPA subcellular structures.

**Original Locations**	**Collapsed Location**
Actin filaments	Actin filaments
Focal adhesion sites	Actin filaments
Centriolar satellite	Centrosome
Centrosome	Centrosome
Aggresome	Cytoplasmic Aggregates
Cytoplasmic bodies	Cytoplasmic Aggregates
Rods & rings	Cytoplasmic Aggregates
Lipid droplets	Cytoplasmic Aggregates
Cytosol	Cytosol
Endoplasmic reticulum	Endoplasmic reticulum
Golgi apparatus	Golgi apparatus
Intermediate filaments	Intermediate filaments
Cleavage furrow	Microtubules
Cytokinetic bridge	Microtubules
Microtubule ends	Microtubules
Microtubules	Microtubules
Midbody	Microtubules
Midbody ring	Microtubules
Mitotic spindle	Microtubules
Mitochondria	Mitochondria
Kinetochore	Nuclear bodies
Nuclear bodies	Nuclear bodies
Nuclear membrane	Nuclear membrane
Nuclear speckles	Nuclear speckles
Nucleoli	Nucleoli
Nucleoli fibrillar center	Nucleoli
Nucleoli rim	Nucleoli
Mitotic chromosome	Nucleoplasm
Nucleoplasm	Nucleoplasm
Cell junctions	Plasma membrane
Plasma membrane	Plasma membrane
Endosomes	Vesicles
Lysosomes	Vesicles
Peroxisomes	Vesicles
Vesicles	Vesicles

Table S2: Primary antibodies targeting metabolic state markers and tubulin, and secondary antibodies used in the 4i experiment

Cycle	Antibody	Concentration	Supplier	Product number
1-3	Rat anti-tubulin IgG2a	2 µg/mL	Invitrogen	MA1-80017
1	Mouse anti-ACACA IgG2a	2 µg/mL	Proteintech	67373-1-IG
2	Mouse anti-PCK2 IgG1	2 µg/mL	Invitrogen	MA5-38591
3	Mouse anti-SOD2 IgG	2 µg/mL	Invitrogen	MA1-106
**Secondary antibodies**				
1-3	Donkey Anti-Rat IgG H&L (Alexa Fluor® 488) preadsorbed	2.5 µg/mL	Abcam	ab150153
1-3	Goat Anti-Mouse IgG H&L (Alexa Fluor® 555) preadsorbed	2.5 µg/mL	Abcam	ab150118
1-3	Goat anti-Rabbit IgG (H+L) Highly Cross-Adsorbed, Alexa Fluor™ 647	2.5 µg/mL	Invitrogen	A21245

Table S3: HPA antibodies targeting metabolic proteins used in the 4i experiment

Protein name	**HPA antibody** (rabbit polyclonal, 2 µg/mL)	Cycle
ECHS1	HPA022476	1
MTHDF1L	HPA029041	1
FDXR	HPA044393	1
ACADS	HPA022271	1
DAGLB	HPA069377	1
GLO1	HPA059791	1
HADHA	HPA015536	1
COX5B	HPA034517	1
COX6C	HPA014295	1
ACADVL	HPA019006	1
PLPP2	HPA058428	1
GPX1	HPA044758	1
L2HGDH	HPA065409	1
PDIA3	HPA002645	1

PNPLA2	HPA062602	1
ACOX3	HPA035840	1
PLPP3	HPA072751	1
GSR	HPA064806	1
ALDH6A1	HPA029075	1
LOXL2	HPA056542	1
ALDH1B1	HPA021037	1
CYP2S1	HPA049227	1
GSS	HPA059315	1
ABCC4	HPA002476	1
IDH3B	HPA049387	1
CYBRD1	HPA014757	1
DEGS1	HPA076422	1
SDHB	HPA002868	1
PHGDH	HPA024031	1
NDUFV3	HPA030427	1
GLRX2	HPA023087	1
GCDH	HPA043252	1
SGPP1	HPA053149	1
GSTP1	HPA019779	1
G6PD	HPA000834	1
RDH14	HPA056686	1
HADH	HPA043888	1
ALOX12	HPA064819	1
PDIA6	HPA034652	1
LBR	HPA062236	1
ELOVL5	HPA047752	1
ACAA1	HPA007244	1
HADHB	HPA066099	1
COX10	HPA032005	1
PRDX5	HPA037915	1

PTGES	HPA045064	2
MPO	HPA061464	2
ALDH4A1	HPA006401	2
DLST	HPA003010	2
MDH1	HPA027296	2
NDUFB5	HPA005640	2
SLCO1A2	HPA071152	2
SMOX	HPA060198	2
ALDH5A1	HPA029716	2
FAR1	HPA017322	2
MECR	HPA028740	2
NDUFB9	HPA042768	2
SLCO1B3	HPA004943	2
TACO1	HPA021643	2
BDH2	HPA036029	2
GLDC	HPA052887	2
MMAA	HPA037361	2
NDUFC1	HPA044556	2
ACSF3	HPA008322	2
AKR1B1	HPA026425	2
BLVRA	HPA019709	2
HPGD	HPA005679	2
NDUFS1	HPA064605	2
AMACR	HPA020912	2
ALDH1A3	HPA046271	2
CYB5R3	HPA001566	2
HSD11B2	HPA056385	2
NDUFA13	HPA041213	2
BAAT	HPA021330	2
ALDH1L1	HPA050139	2
DBT	HPA026485	2

IDH2	HPA007831	2
NDUFAB1	HPA054364	2
FKBP8	HPA045177	2
HAO1	HPA072442	2
ALDH1L2	HPA039481	2
LDHA	HPA075026	2
NDUFB1	HPA063737	2
NDUFV1	HPA075051	2
MAOA	HPA054807	2
ALDH3A1	HPA063783	2
DHRS2	HPA053915	2
AK3	HPA063324	2
NDUFB4	HPA051739	2
NQO1	HPA007308	2
NQO2	HPA021332	3
CRYZ	HPA021921	3
SPR	HPA039505	3
ATP5PB	HPA057347	3
CES1	HPA046717	3
CASP1	HPA003056	3
PDHB	HPA036745	3
DHCR7	HPA044280	3
TBCB	HPA041428	3
ATP5PD	HPA042777	3
ACO1	HPA019371	3
NR2C2	HPA006313	3
PECR	HPA021593	3
DIO3	HPA073684	3
TM7SF2	HPA072704	3
ATP5PF	HPA031069	3
TKT	HPA029481	3

RELA	HPA063461	3
PGLS	HPA042032	3
ELOVL1	HPA056557	3
COX4I1	HPA002485	3
MT-CYB	HPA068400	3
NFKBIA	HPA029207	3
RDH13	HPA042006	3
FDFT1	HPA008874	3
COX6B1	HPA004192	3
NDUFA7	HPA059251	3
PPARG	HPA063663	3
RLBP1	HPA044083	3
FMO5	HPA012373	3
ATP5ME	HPA035010	3
PPA1	HPA020096	3
NFKB1	HPA027305	3
IL33	HPA022899	3
TXNRD1	HPA001395	3
NOS1	HPA058312	3
PRUNE1	HPA028411	3
SREBF1	HPA043878	3
OCLN	HPA005933	3
AKR1A1	HPA019649	3
PTGS1	HPA002834	3
ATP5MJ	HPA058978	3
UQCRC1	HPA003525	3
GSDMD	HPA044487	3
RRM1	HPA057265	3
