# Supplementary material for: Dissecting autonomous enzymatic variability in single cells
Source: Nat Commun. 2026 Jul 2;17:5788. doi: 10.1038/s41467-026-74172-z (PMC13328634; doi:10.1038/s41467-026-74172-z)
Supplement: Supplementary file 2 — Reporting Summary [file 41467_2026_74172_MOESM2_ESM.pdf]

nature portfolio

Corresponding author(s): Emma Lundberg

Last updated by author(s): 2026/03/09

Reporting Summary

Nature Portfolio wishes to improve the reproducibility of the work that we publish. This form provides structure for consistency and transparency in reporting. For further information on Nature Portfolio policies, see our Editorial Policies and the Editorial Policy Checklist.

Statistics

For all statistical analyses, confirm that the following items are present in the figure legend, table legend, main text, or Methods section.

n/a	Confirmed	
☐	☒	The exact sample size (*n*) for each experimental group/condition, given as a discrete number and unit of measurement
☐	☒	A statement on whether measurements were taken from distinct samples or whether the same sample was measured repeatedly
☐	☒	The statistical test(s) used AND whether they are one- or two-sided<br>*Only common tests should be described solely by name; describe more complex techniques in the Methods section.*
☐	☒	A description of all covariates tested
☐	☒	A description of any assumptions or corrections, such as tests of normality and adjustment for multiple comparisons
☐	☒	A full description of the statistical parameters including central tendency (e.g. means) or other basic estimates (e.g. regression coefficient) AND variation (e.g. standard deviation) or associated estimates of uncertainty (e.g. confidence intervals)
☐	☒	For null hypothesis testing, the test statistic (e.g. *F*, *t*, *r*) with confidence intervals, effect sizes, degrees of freedom and *P* value noted<br>*Give P values as exact values whenever suitable.*
☒	☐	For Bayesian analysis, information on the choice of priors and Markov chain Monte Carlo settings
☒	☐	For hierarchical and complex designs, identification of the appropriate level for tests and full reporting of outcomes
☐	☒	Estimates of effect sizes (e.g. Cohen's *d*, Pearson's *r*), indicating how they were calculated

Our web collection on statistics for biologists contains articles on many of the points above.

Software and code

Policy information about availability of computer code

Data collection	The majority of data was mined from public and published datasets, 4i data was using Harmony 5.2 (Revvity) on the Operetta Phenix High-Content Screening System.
Data analysis	4i analysis was performed in CellProfiler v4.2.6 and Python 3.10, all other analysis in Python v3.7.1 using pandas v1.3.5, scipy v1.7.3, numpy 1.26.5, matplotlib v3.5.3, seaborn v0.11.2, statsmodels v0.13.2 if not otherwise indicated. All custom analysis and visualization code and packages used is public on Github (https://github.com/CellProfiling/MetabolismManuscript/).

For manuscripts utilizing custom algorithms or software that are central to the research but not yet described in published literature, software must be made available to editors and reviewers. We strongly encourage code deposition in a community repository (e.g. GitHub). See the Nature Portfolio guidelines for submitting code & software for further information.

Data

Policy information about availability of data

All manuscripts must include a data availability statement. This statement should provide the following information, where applicable:

- Accession codes, unique identifiers, or web links for publicly available datasets
- A description of any restrictions on data availability
- For clinical datasets or third party data, please ensure that the statement adheres to our policy

Images are publicly available on the HPA website (www.proteinatlas.org), and single-cell transcriptomic data are available in GEO SRA project GSE146773.

Uncompressed images for the cell cycle-resolved imaging proteomic dataset were annotated using IDR metadata templates and deposited in the BioImage Archive (accession S-BIAD34, https://www.ebi.ac.uk/biostudies/BioImages/studies/S-BIAD34). 4i imaging data was deposited in the BioImage Archive (accession BIAD2984; https://www.ebi.ac.uk/biostudies/BioImages/studies/S-BIAD2984). Source data underlying figures 1a,c,e,f; 2b,c,d; 3b; 5c and extended data figures 1a,b,c,d,f,g; 2a,b,f,g,h; 6a,b,c is provided in the Source Data File. All additional data is provided in the Supplementary Data on figshare (accession code 31566880, DOI: 10.6084/m9.figshare.31566880).

Research involving human participants, their data, or biological material

Policy information about studies with human participants or human data. See also policy information about sex, gender (identity/presentation), and sexual orientation and race, ethnicity and racism.

Reporting on sex and gender	not applicable to this study
Reporting on race, ethnicity, or other socially relevant groupings	not applicable to this study
Population characteristics	not applicable to this study
Recruitment	not applicable to this study
Ethics oversight	not applicable to this study

Note that full information on the approval of the study protocol must also be provided in the manuscript.

Field-specific reporting

Please select the one below that is the best fit for your research. If you are not sure, read the appropriate sections before making your selection.

☒ Life sciences ☐ Behavioural & social sciences ☐ Ecological, evolutionary & environmental sciences

For a reference copy of the document with all sections, see nature.com/documents/nr-reporting-summary-flat.pdf

Life sciences study design

All studies must disclose on these points even when the disclosure is negative.

Sample size	The study mainly used data from public repositories including the Human Protein Atlas, OpenCell, BioPlex or other published datasets. In the 4i experiment, hundreds of cells were collercted per condition allowing for robust statistical analysis.
Data exclusions	In the 4i experiment, incomplete cells touching the border of the image and small cells (<1500 pixels) were excluded from the analysis.
Replication	The study mainly used data from public repositories. In the 4i experiment, we observed high reproducibility across replicates and no batch effects were observed (Extended Data Fig. 4a,b)
Randomization	The study mainly used data from public repositories. In the 4i experiment, images were acquired at the same positions in each well without manual intervention.
Blinding	Data processing and analysis was automated without manual intervention.

Reporting for specific materials, systems and methods

We require information from authors about some types of materials, experimental systems and methods used in many studies. Here, indicate whether each material, system or method listed is relevant to your study. If you are not sure if a list item applies to your research, read the appropriate section before selecting a response.

Materials & experimental systems

n/a	Involved in the study	
☐	☒	Antibodies
☐	☒	Eukaryotic cell lines
☒	☐	Palaeontology and archaeology
☒	☐	Animals and other organisms
☒	☐	Clinical data
☒	☐	Dual use research of concern
☒	☐	Plants

Methods

n/a	Involved in the study	
☒	☐	ChIP-seq
☒	☐	Flow cytometry
☒	☐	MRI-based neuroimaging

Antibodies

Antibodies used	The study is largely based on data from the Human Protein Atlas database (v23). For the 4i experiment 4 additional antibodies were used, Rat anti-tubulin IgG2a (Invitrogen MA1-80017), Mouse anti-ACACA IgG2a (Proteintech 67373-1-IG), Mouse anti-PCK2 IgG1 (Invitrogen MA5-38591) Mouse anti-SOD2 IgG (Invitrogen MA1-106).
Validation	All HPA antibodies are validated for use in immunofluorescence assays and the validation data is publicly available on proteinatlas.org. For commercial antibodies, the validation data for immunofluorescence assays in human cell lines is supplied by vendors. To ensure the specificity of the antibodies to be included in the experiemnt, each antibody was validated by indirect immunofluorescence staining.

Eukaryotic cell lines

Policy information about cell lines and Sex and Gender in Research

Cell line source(s)	HEK293T (ATCC CRL-3216) cell were used in mNG tagging experiment. U2OS cells (HTB-96) were used for 4i imaging.
Authentication	All cell lines were authenticated as part of the Human Protein Atlas database ((https://www.proteinatlas.org/learn/cellines)
Mycoplasma contamination	Cell lines were routinely tested for mycoplasma contamination.
Commonly misidentified lines (See ICLAC register)	None of the cell lines used for mNG tagging or 4i are commonly misidentified cell lines.

Plants

Seed stocks	*Report on the source of all seed stocks or other plant material used. If applicable, state the seed stock centre and catalogue number. If plant specimens were collected from the field, describe the collection location, date and sampling procedures.*
Novel plant genotypes	*Describe the methods by which all novel plant genotypes were produced. This includes those generated by transgenic approaches, gene editing, chemical/radiation-based mutagenesis and hybridization. For transgenic lines, describe the transformation method, the number of independent lines analyzed and the generation upon which experiments were performed. For gene-edited lines, describe the editor used, the endogenous sequence targeted for editing, the targeting guide RNA sequence (if applicable) and how the editor was applied.*
Authentication	*Describe any authentication procedures for each seed stock used or novel genotype generated. Describe any experiments used to assess the effect of a mutation and, where applicable, how potential secondary effects (e.g. second site T-DNA insertions, mosiacism, off-target gene editing) were examined.*
