# Supplementary material for: Dissecting autonomous enzymatic variability in single cells
Source: Nat Commun. 2026 Jul 2;17:5788. doi: 10.1038/s41467-026-74172-z (PMC13328634; doi:10.1038/s41467-026-74172-z)
Supplement: Supplementary file 4 — Description of Additional Supplementary Files [file 41467_2026_74172_MOESM4_ESM.pdf]

Supplementary Data

Supplementary Data 1
List of enzymes from Human1 database along with pathway annotations

Supplementary Data 2
Export from HPA Subcellular Section (v23) including protein location and expression heterogeneity information for 13147 proteins

Supplementary Data 3
Pathway centric subcellular localisation information.

Supplementary Data 4
Results from annotation from Reicher et al. (DOI: 10.1038/s41556-024-01407-w)

Supplementary Data 5
Comparision with murine liver zonation of metabolic enzymes by Rosenberger et al. (DOI: 10.1038/s41592-023-02007-6)

Supplementary Data 6
Results from upstream kinase analysis
Supplementary Data 7
Single-cell features extracted in 4i experiment

Supplementary Data 8
Experimentally verified location information from UniProt

Supplementary Data 9
Protein-protein interaction database curated from open-source AP-MS datasets
