# Supplementary material for: Dissecting autonomous enzymatic variability in single cells
Source: Nat Commun. 2026 Jul 2;17:5788. doi: 10.1038/s41467-026-74172-z (PMC13328634; doi:10.1038/s41467-026-74172-z)
Supplement: Supplementary file 3 — Transparent Peer Review file [file 41467_2026_74172_MOESM3_ESM.pdf]

nature portfolio

Peer Review File

Dissecting autonomous enzymatic variability in single cells

Corresponding Author: Professor Emma Lundberg

This file contains all reviewer reports in order by version, followed by all author rebuttals in order by version.

Version 0:

Reviewer comments:

Reviewer #1

(Remarks to the Author)
Gnann et al. present a manuscript interrogating the variability of metabolic enzymes in multiplex-IF data derived from cell lines, as documented in the Human Protein Atlas (HPA). The study emphasizes the localization of enzymes within cellular organelles and compartments.

The manuscript addresses a cutting-edge and understudied question, one that remains relatively unexplored due to the lack of methods for comprehensive analysis. I commend the authors for reusing HPA data, which provides a large, validated resource for such investigations.

The authors' analysis is thorough and rigorous. Their interpretation of the results is well-supported by evidence and clearly articulated.

The manuscript is well-written, engaging, and easy to follow. I particularly appreciate the authors' use of numerous examples featuring well-known enzymes and their inclusion of IF images, which make this systems biology work more accessible and thought-provoking.

I do not have any major critical comments and believe the manuscript can be accepted largely as is. However, I offer a few suggestions that could enhance the manuscript—though these are entirely at the authors' discretion.

1. Nuclear Metabolism: There has been growing interest in nuclear metabolism, including the surprising detection of TCA cycle activity within the nucleus (Li et al Nat Comms 2022 https://www.nature.com/articles/s41467-022-35199-0, Kafkia et al Sci Adv 2022 https://www.science.org/doi/10.1126/sciadv.abq5206). Could the authors identify evidence for this phenomenon in the HPA data? Complementing these findings with an analysis of single-cell heterogeneity in nuclear metabolism would add significant depth to the discussion.

2. Exploring Factors Associated with Heterogeneity: Since heterogeneity is central to the manuscript, the authors could extend their analysis to investigate factors that drive the reported variability. While Fig. 2d is an excellent starting point, it simplifies the underlying complexity. Could the authors conduct an association analysis to identify key factors (e.g. GO-terms) that are associated or explain variability levels most?

3. Macro-Spatial Factors of Heterogeneity: Are the localization and intensities of enzymes in neighboring cells statistically independent? Alternatively, do they depend on the cells being direct neighbors? Examining spatial dependencies could offer additional insights into the heterogeneity reported.

Here are some minor comments comments which can be addressed:
* Multi-Localizing Enzymes: Can the authors comment on or refer to prior work that confirms the IF detection is not an artifact of background fluorescence, particularly in dense compartments like the nucleus?

* Fig. 2 Caption: The caption for Fig. 2 needs correction—it currently omits Fig. 2c and mislabels other panels.

* Define CCD and Non-CCD: Please provide definitions for “CCD” and “non-CCD” for clarity.

* Terminology Consistency: The terms “moonlighting” and “multifunctional” are used inconsistently, as are “variability” and

"heterogeneity."

Reviewer #2

(Remarks to the Author)
The results about metabolic enzymes are interesting, however, the images of metabolic enzymes are not from the "same single cells". Thus, the findings presented are ill-defined.

The suggestion is to use CyCIF, CODEX, IMC, MIBI, or similar method to validate the biological conclusions.

Also, it would be important to remove claims about spatial proteomics - which is nowadays referring to the "same single cells" not a historical library of datasets from ancient images.

Reviewer #3

(Remarks to the Author)
This study presented results from an analysis of the metabolic proteome data in the HPA (HPAv23 Subcellular Section, 17,180 IF images). This is a comprehensive, unique resource for studying spatial biology of cellular metabolism. The study generated some interesting biological insights (e.g., localization, dynamics, multi-localization of some enzyme expressions) and verified/expanded some previous understanding on spatial metabolism. While those were exciting, insightful, the manuscript was written in a highly descriptive way. The data analysis and the methods were not described in sufficient details to establish statistical rigor of the analysis, which I think is important when it comes to IF image data analysis and can significantly affect conclusions or accuracy in this study.
Also, the work is not validated/compared (at least partially) against orthogonal data/technologies that directly inform spatial metabolism (e.g., MALDI), leaving one wondering how precise/informative the observations/conclusions are.
Comments:
Generally, when a concept is defined, it is necessary to quantify it with a mathematical/statistical metric, and describe what kind of test (and why that) was used to determine the value. For example:
Page 4. "metabolic enzymes across all pathway groups exhibit a significantly higher degree of heterogeneous expression ". It is unclear how this heterogeneity (related to dynamics) is quantified and compared against the background. What was the metric used, and what was the test used to derive the conclusion (heterogeneous vis non-heterogeneous). Missing methodological descriptions.
Page 4, "we performed a gene ontology (GO)-term enrichment analysis of variably and stably expressed enzymes against the background of the entire metabolic proteome". How were variably and stably expressed enzymes defined from the data? What were the quantification metric and cutoff to classify them?
Page 6, "more than half of all metabolic proteins are multi localizing ". Only a brief description of "multi-localizing" was found in Methods with no detailed description on quantification. This is insufficient and likely inaccurate, as the imaging data can have various type/level of noise, which can significantly affect the conclusions or textual description.

Some important methodological details are missing:
Page 3, "To quantify the similarity of subcellular distribution for different sets of metabolic enzymes, we embedded the spatial information from the images into a compressed representation using a machine learning approach (Methods).30" I could not find the corresponding description in Methods. Reference [30] did not describe how to embed spatial information with the protein expressions onto high dimensional or UMAP representations shown in Fig. 1d and 1g.
Minor comments:
Page 3: "Of these genes, we have measured the subcellular distribution for 2,126 proteins". How many genes these 2,126 proteins correspond to?
Fig. 1g, in the color legend, the colored dots and the text lables are unaligned.
Fig. 2b, the Y axis label "scv" needs to be spelled out.
Fig.2e, "HPA" appeared twice in the column labels.
Fig. 2f, "mNG" appeared twice in the column labels.
Fig. 4 is largely illegible, showing largely just the variability. I think it needs to be presented in some other ways.

Reviewer #4

(Remarks to the Author)
I co-reviewed this manuscript with one of the reviewers who provided the listed reports. This is part of the Nature Communications initiative to facilitate training in peer review and to provide appropriate recognition for Early Career Researchers who co-review manuscripts.

Reviewer #5

(Remarks to the Author)
I co-reviewed this manuscript with one of the reviewers who provided the listed reports. This is part of the Nature Communications initiative to facilitate training in peer review and to provide appropriate recognition for Early Career Researchers who co-review manuscripts.

Version 1:

Reviewer comments:

Reviewer #1

(Remarks to the Author)
The revision has addressed my comments, improved the manuscript, and made it stronger.

It is exciting to see the newly added results on the subcellular localization of enzymes in Extended Data Fig. 8, and in particular, the breakdown by metabolic pathways in Extended Data Fig. 7. The finding that energy metabolism is localized mainly to the mitochondria (Extended Data Fig. 7d) provides strong validation of this approach. I believe these data will be of high value to the metabolism community. If possible, it would be very helpful to include a summary of Extended Data Fig. 7 broken down by subcellular compartments (i.e., for each compartment, show which metabolic pathways are represented there). Alternatively, this could be considered for inclusion in a future paper or on the HPA website.

Reviewer #2

(Remarks to the Author)
Authors have provided answers to the concerns raised in the previous version. Unfortunately, the revised version is premature in terms of achieving true variability of metabolic enzymes.

1. The presented 4i data only multiplex 7 markers - nothing close to 30-50 markers conventionally used in CODEX, MIBI, CycIF, and similar. The 7 marker can also be achieved by Opal dyes without multiplexing. Then the authors changed the protein targets for various 7-marker panels, but it would not necessarily address the concern of the lack of true "same single cell" claims.

2. The accumulated library of these images have been reported in so many other publications for this cell image database. This paper does not yet provide novelty on top of what was published before from the same group and others using the proteinatlas.org website. Not convinced that the results with "high dimensional" feature analysis could be a reliable source. The definition of "location" in one cell versus another single cell could be largely different - and thus data integration in this paper is indeed problematic.

Reviewer #3

(Remarks to the Author)
The authors have addressed my comments. I have no further comments.

Reviewer #4

(Remarks to the Author)
I co-reviewed this manuscript with one of the reviewers who provided the listed reports. This is part of the Nature Communications initiative to facilitate training in peer review and to provide appropriate recognition for Early Career Researchers who co-review manuscripts.

Reviewer #5

(Remarks to the Author)
I co-reviewed this manuscript with one of the reviewers who provided the listed reports. This is part of the Nature Communications initiative to facilitate training in peer review and to provide appropriate recognition for Early Career Researchers who co-review manuscripts.

Version 2:

Reviewer comments:

Reviewer #2

(Remarks to the Author)
Authors provided responses to my questions, but they provide "Shortcuts" to avoid additional experiments.

1. Multiplexing metabolic enzymes are currently established in a few recent papers:
https://www.researchsquare.com/article/rs-6695435/v1, https://pubmed.ncbi.nlm.nih.gov/32868913/,
https://www.liebertpub.com/doi/10.1089/genbio.2023.0029.

These papers optimized up to 100 antibodies for cell types and metabolic enzyme targets- and their insights from metabolic enzyme variability is translational and much more interesting than this current paper. They use the "same tissue" and generate insights about clinical end goals. The current study uses previously genereated data from years ago cells in a

culture - which is non-physiological.

4i and Cycif experiments are inexpensive. The authors have more than 12,000 antibodies already in their hands when the data was generated from proteinatlas.org. the rest is a few chemical from sigma (less than a few hundreds dollars).

2. To repeat metabolic enzyme variabilty in different cell populations, the recent paper on cell perception https://www.science.org/doi/full/10.1126/science.abf4062 demonstrates that even single cells in cultures located in different colony positions perceive growth signals differently. I cannot imagine all these different experiments and their differences and how they lead to subtle and gross changes in metabolic enzyme states.

Metabolic enzymes are pretty interesting and very important but this study may lead to risky and non physiological patterns (that might not be real) and create hallucination problem in single cell biology.

Open Access This Peer Review File is licensed under a Creative Commons Attribution 4.0 International License, which permits use, sharing, adaptation, distribution and reproduction in any medium or format, as long as you give appropriate credit to the original author(s) and the source, provide a link to the Creative Commons license, and indicate if changes were made.
In cases where reviewers are anonymous, credit should be given to 'Anonymous Referee' and the source.
The images or other third party material in this Peer Review File are included in the article's Creative Commons license, unless indicated otherwise in a credit line to the material. If material is not included in the article's Creative Commons license and your intended use is not permitted by statutory regulation or exceeds the permitted use, you will need to obtain permission directly from the copyright holder.
To view a copy of this license, visit https://creativecommons.org/licenses/by/4.0/

Rebuttal to reviewer comments

Comments from reviewer #1:

It is exciting to see the newly added results on the subcellular localization of enzymes in Extended Data Fig. 8, and in particular, the breakdown by metabolic pathways in Extended Data Fig. 7. The finding that energy metabolism is localized mainly to the mitochondria (Extended Data Fig. 7d) provides strong validation of this approach. I believe these data will be of high value to the metabolism community. If possible, it would be very helpful to include a summary of Extended Data Fig. 7 broken down by subcellular compartments (i.e., for each compartment, show which metabolic pathways are represented there). Alternatively, this could be considered for inclusion in a future paper or on the HPA website.

We want to thank the reviewer for their positive comments on our work as well as constructive feedback and suggestions.

Fig. 1c contains a summary on subcellular localization of metabolic pathways and includes a quantitative analysis of which pathways groups are overrepresented in which subcellular compartment. To allow for easier data mining of this data, as suggested by the reviewer, we have now included an additional Supplementary Table 3. For each pathway and pathway group, we provide the number of enzymes in each subcellular compartment as well as the corresponding gene names. We hope that this resource will be helpful to the community.

Comments from reviewer #2:

1. The presented 4i data only multiplex 7 markers - nothing close to 30-50 markers conventionally used in CODEX, MIBI, CycIF, and similar. The 7 marker can also be achieved by Opal dyes without multiplexing. Then the authors changed the protein targets for various 7-marker panels, but it would not necessarily address the concern of the lack of true "same single cell" claims.

We thank the reviewer for expressing their concern about our data, and have now updated the manuscript to clarify the language to avoid misunderstandings.

The multiplexed experiment in question has validated our claims about metabolic heterogeneity, which is what has been requested in the previous round of revisions. As outlined in the last rebuttal, we make no claims in measuring all the target enzymes in the same single cell. In fact, such multiplexing is not feasible with the state-of-the-art technologies due to:

1) Lack of commercially available antibodies compatible with the proposed imaging technologies. Panels provided by Akoya and similar companies include at most 9 metabolic proteins, whereas we are reporting on 135 enzymes in the multiplexed experiment. Such panels usually aim to identify cell *types*, not (metabolic) cell *states.* As a side note, some of the commercially available “cell type” markers used in these panels (CD38, MPO) show up in our study as variable metabolic enzymes *within a cell type / line*. We believe it is crucial to communicate this information to the broader community,

as these findings raise questions about the reliability of using these proteins as immutable cell type markers, especially in new tissue contexts.

2) Extremely high costs of developing such a panel in house. From our extensive experience in this matter[1–5], we estimate the costs of conjugating the antibodies, validating them and running the experiments to be over 1 million USD. Additionally, the process of antibody conjugation and validation alone can take up to 1 month *per target.*
3) Questionable quality of the samples and stainings in the experiments relying on cyclic immunofluorescent protocols (4i or CycIF) past the first couple of cycles. Using the validated HPA rabbit antibodies would allow only one target to be imaged per cycle.The authors of 4i generally warn about the sample degradation and changes in antibody staining past 20 cycles [6]. The latest record, to our knowledge, is 95 cycles (Kuehl et al., Nature, 2025[7]), which would still yield a lower number of targets than what we have achieved with our experimental setup.

While we agree with the reviewer that it would be extremely valuable to have this kind of data from the exact same cells, we do not think it is technically possible with the state-of-the-art technologies. Such experiments are definitely worth pursuing in the future, but they require novel technological breakthroughs and are way beyond the scope of the current paper.

We reiterated that our results are not derived from the same single cells on two occasions in our conclusion to avoid misinterpretation of our findings and claims. The modified text of the manuscript reads as follows:

"Consequently, our single-cell proteome-wide metabolic map, although not obtained from the same individual cells, offers an important approximation of cellular metabolic heterogeneity and provides a valuable step towards improved metabolic modeling which may change our understanding of the metabolic regulation of cell types."

An immense number of possible metabolic states are possible, given the 800 metabolic enzymes that we show to have variable expression in separate experiments and the continuum of metabolic states that we observed using co-stainings for up to 6 enzymes using 4i. Even so, established biochemical frameworks describe a relatively small number of high-level, often mutually exclusive metabolic states (switches), such as glycolysis-dominated or respiration-dominated energy production. Co-regulated enzymatic activity within a metabolic pathway can lead to co-variation of enzyme expression. In this case, it may be possible to pinpoint highly variable metabolic enzymes that are markers for distinct high-level metabolic states within the cell population. However, this co-variation is not absolute; one enzyme can show relatively higher expression than other enzymes in the same pathway if its product is required for another metabolic pathway or if it is performing several different functions. Mapping such variations in the same single cells using multiplexed imaging strategies as proposed in this study, or single cell mass spectrometry-based technologies may reveal the complex landscape of more granular metabolic phenotypes, including the connections, dependencies and compensatory interactions between different metabolic pathways."

2. The accumulated library of these images have been reported in so many other publications for this cell image database.This paper does not yet provide novelty on top of what was published before from the same group and others using the proteinatlas.org website. Not convinced that the results with "high dimensional" feature analysis could be a reliable source. The definition of "location" in one cell versus another single cell could be largely different - and thus data integration in this paper is indeed problematic.

We would like to clarify that data integration approaches [8–10] and image vision model-based analysis[11–15] described in our manuscript are both well established in the field. The approaches we use have been extensively tested, characterized and previously published in high-ranking peer-reviewed journals both by our own group and by other colleagues.

We have to respectfully disagree with the claim of the lack of novelty. Re-analysis of the existing large datasets is common in biology and can provide valuable insights. Our study is the first one to (a) focus on metabolic enzymes and the heterogeneity of their expression; (b) bring together the heterogeneous expression and the multilocalization aspects. These aspects have been studied separately, but never analyzed within the same dataset, and we see this as one of the strengths of our approach.

Additionally, our study includes a number of experimental datasets apart from the atlas data: staining of the FUCCI cell line to explore the connection between metabolic enzyme expression and the cell cycle; clonal expansion experiments of cells with mNG-tagged enzymes; the multiplexed 4i dataset; the compartment-resolved analysis protein-protein interaction data. We would like to emphasize that the latter, for example, has led to us formulating a scientifically verifiable hypothesis for a new biological function of P4HA2 *specifically in the nucleus* and only in the subset of cells. As such, we strongly believe that our study has sufficient methodological and biological novelty beyond the information currently available in the literature.

References

1. Quardokus, E. M. *et al.* Organ Mapping Antibody Panels: a community resource for standardized multiplexed tissue imaging. *Nat. Methods* **20**, 1174–1178 (2023).
2. Mardamshina, M. *et al.* Multiplexed Deep Visual Proteomics Unveils Spatial Heterogeneity and Rare Endocrine States in Human Adult Pancreatic Islets. 2025.04.27.650857 Preprint at https://doi.org/10.1101/2025.04.27.650857 (2025).
3. Mereu, E. *et al.* Latent plasticity of the human pancreas across development, health, and disease. 2025.10.01.679230 Preprint at https://doi.org/10.1101/2025.10.01.679230 (2025).
4. Sariyar, S. *et al.* High-parametric protein maps reveal the spatial organization in early-developing human lung. *Nat. Commun.* **15**, 9381 (2024).
5. Sun, H. *et al.* Flexible and robust cell type annotation for highly multiplexed tissue images. *bioRxiv* 2024.09.12.612510 (2024) doi:10.1101/2024.09.12.612510.
6. Kramer, B. A., Del Castillo, J. S., Pelkmans, L. & Gut, G. Iterative Indirect Immunofluorescence Imaging (4i) on Adherent Cells and Tissue Sections. *Bio-Protoc.* **13**, e4712 (2023).
7. Kuehl, M. *et al.* Pathology-oriented multiplexing enables integrative disease mapping. *Nature* **644**, 516–526 (2025).
8. Kramer, B. A., Sarabia Del Castillo, J. & Pelkmans, L. Multimodal perception links cellular state to decision-making in single cells. *Science* **377**, 642–648 (2022).
9. Stossi, F. *et al.* SPACe: an open-source, single-cell analysis of Cell Painting data. *Nat. Commun.* **15**, 10170 (2024).
10. Chandrasekaran, S. N. *et al.* JUMP Cell Painting dataset: morphological impact of 136,000 chemical and genetic perturbations. 2023.03.23.534023 Preprint at https://doi.org/10.1101/2023.03.23.534023 (2023).
11. Doron, M. *et al.* Unbiased single-cell morphology with self-supervised vision transformers. *bioRxiv* 2023.06.16.545359 (2023) doi:10.1101/2023.06.16.545359.

12. Ouyang, W. *et al.* Analysis of the Human Protein Atlas Image Classification competition. *Nat. Methods* **16**, 1254–1261 (2019).
13. Le, T. *et al.* Analysis of the Human Protein Atlas Weakly Supervised Single-Cell Classification competition. *Nat. Methods* **19**, 1221–1229 (2022).
14. Cho, N. H. *et al.* OpenCell: proteome-scale endogenous tagging enables the cartography of human cellular organization. *Science* **375**, eabi6983 (2022).
15. Kobayashi, H., Cheveralls, K. C., Leonetti, M. D. & Royer, L. A. Self-supervised deep learning encodes high-resolution features of protein subcellular localization. *Nat. Methods* **19**, 995–1003 (2022).

Authors response to reviewers comments

We are grateful to all reviewers for their constructive feedback and the time and effort dedicated to evaluating our work. Their valuable insights have helped us to significantly improve the manuscript. We have diligently addressed each comment and implemented suggestions and clarifications in the revised manuscript file (updated text in blue font color). In response to the reviewers' comments, we have clarified our method section, performed additional 4i multiplexed imaging experiments to more accurately profile metabolic states, provided new analysis, and updated several figures. Please see our point-by-point responses below for details.

Reviewer #1 (Remarks to the Author):

Gnann et al. present a manuscript interrogating the variability of metabolic enzymes in multiplex-IF data derived from cell lines, as documented in the Human Protein Atlas (HPA). The study emphasizes the localization of enzymes within cellular organelles and compartments.

The manuscript addresses a cutting-edge and understudied question, one that remains relatively unexplored due to the lack of methods for comprehensive analysis. I commend the authors for reusing HPA data, which provides a large, validated resource for such investigations.

The authors' analysis is thorough and rigorous. Their interpretation of the results is well-supported by evidence and clearly articulated.

The manuscript is well-written, engaging, and easy to follow. I particularly appreciate the authors' use of numerous examples featuring well-known enzymes and their inclusion of IF images, which make this systems biology work more accessible and thought-provoking.

I do not have any major critical comments and believe the manuscript can be accepted largely as is. However, I offer a few suggestions that could enhance the manuscript—though these are entirely at the authors' discretion.

We are grateful to Reviewer 1 for their comprehensive review and appreciate their constructive comments and well-structured feedback. The suggestions to focus on the biology of multilocalizing and heterogeneously expressed proteins in more detail have strengthened the manuscript.

1. Nuclear Metabolism: There has been growing interest in nuclear metabolism, including the surprising detection of TCA cycle activity within the nucleus (Li et al Nat Comms 2022 https://www.nature.com/articles/s41467-022-35199-0, Kafkia et al Sci Adv 2022 https://www.science.org/doi/10.1126/sciadv.abq5206). Could the authors identify evidence for this phenomenon in the HPA data? Complementing these findings with an analysis of single-cell heterogeneity in nuclear metabolism would add significant depth to the discussion.

We appreciate the reviewer's insightful comment on the role of mitochondrial enzymes and metabolites in the nucleus, which underscores the biological relevance of single-cell heterogeneity and protein multilocalization. In response, we confirmed that 29% of TCA cycle enzymes (12 of 41) in the HPA dataset localize to both the nucleus and either the mitochondria or cytosol. We have expanded the section on protein multilocalization

and added a new supplementary figure (Extended Data Fig. 8a, b) to address this point in greater detail. We agree this addition strengthens the manuscript by providing a compelling example of why large-scale profiling of protein heterogeneity and localization is critical for understanding cellular function.

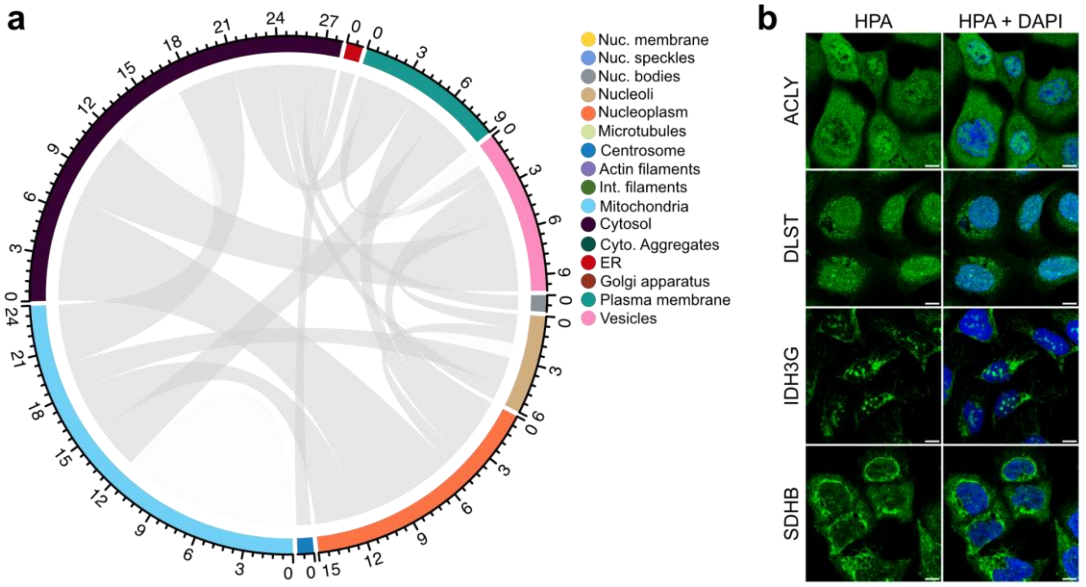

Extended Data Fig. 8: Multifunctional proteins. **a,** Circos plot for all TCA cycle proteins with the identified proteins of each compartment presented and sorted by meta-compartments (cytoplasm = red, secretory pathway = green, nucleus = blue). Multilocalizing proteins are connected by a line (secondary colors represent multilocalization across metacompartments). The plot shows connections among metabolic proteins per pathway group in the HPA dataset. **b)** Example images for TCA cycle enzymes with additional nuclear localization (blue = DAPI, green = protein of interest, scale bar corresponds to 10 µm).

2. Exploring Factors Associated with Heterogeneity: Since heterogeneity is central to the manuscript, the authors could extend their analysis to investigate factors that drive the reported variability. While Fig. 2d is an excellent starting point, it simplifies the underlying complexity. Could the authors conduct an association analysis to identify key factors (e.g. GO-terms) that are associated or explain variability levels most?

We thank the reviewer for this important comment. We acknowledge that our study does not directly address the origins of metabolic heterogeneity, beyond identifying a small subset of enzymes regulated by the cell cycle. Our analysis relies on a binary classification of protein variability (variable vs. non-variable). As shown in Fig. 2d, we performed GO term enrichment analysis to explore biological functions linked to heterogeneous enzyme expression and highlighted that this heterogeneity spans all metabolic pathways and groups (Figs. 4 and 2b). In response to the reviewer's suggestion, we further conducted an association analysis using GO Biological Process terms to identify potential drivers of protein expression variability. However, the predictive power was limited (F1 score = 0.353), likely due to class imbalance across GO terms. Nonetheless, we have included this analysis in the revised manuscript (Extended Data Figure 2c). While we fully agree with the reviewer that understanding the factors that drive the reported variability through perturbation experiments would be very interesting, we believe it is beyond the scope of this study.

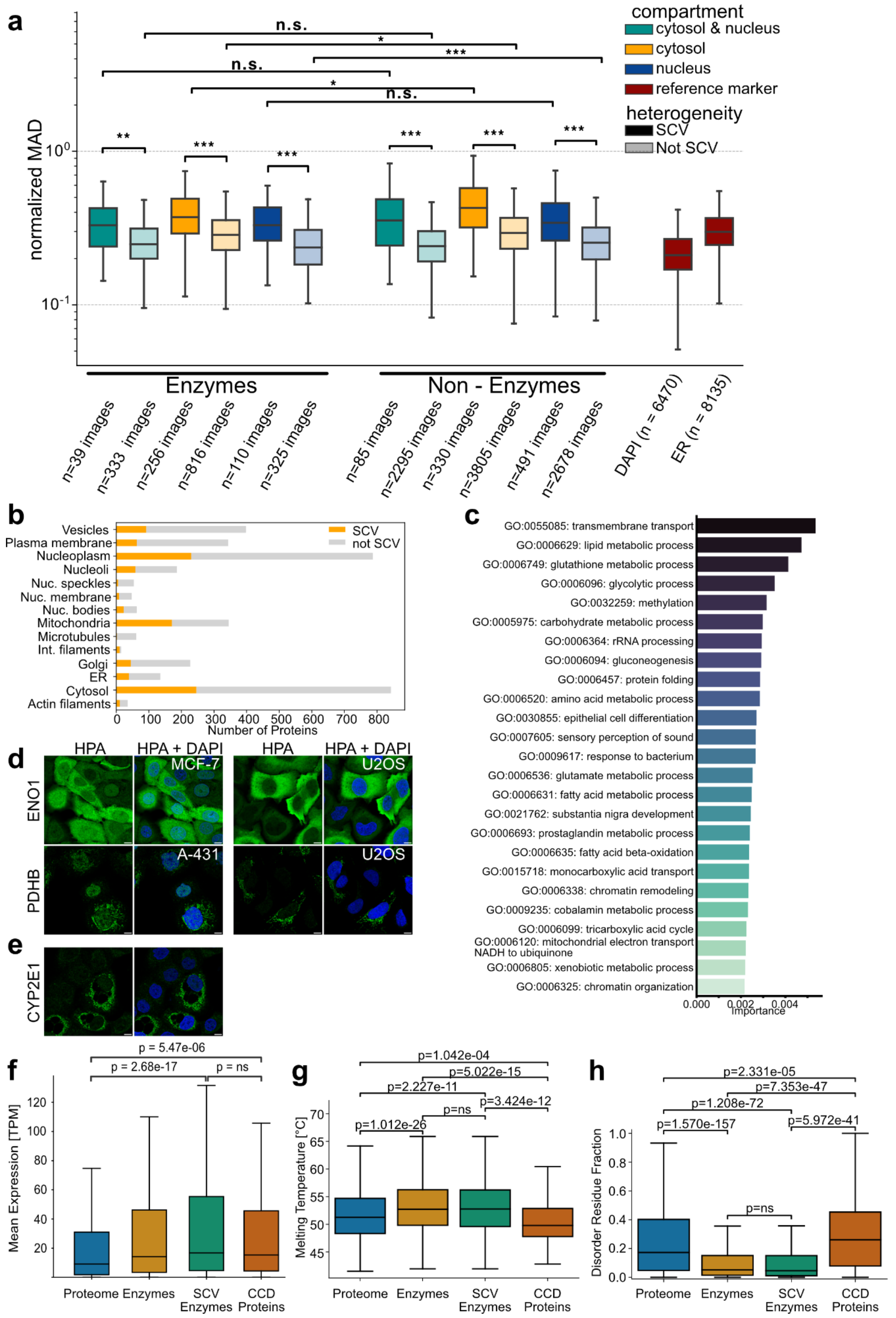

Extended Data Fig. 2: The variable metabolic proteome in the HPA Subcellular Section

a, The normalized median absolute deviation was calculated between individual cells in the same image as a measure of protein expression variability for different groups of proteins (enzymes and proteins in different cellular compartment that are variably expressed or not based on manual annotations from HPAv23. **b,** Degree of variability across cellular localizations for the metabolic proteome. **c,** The predictive power of GO terms (Biological Process) is low for the prediction of protein expression variability. However, the predictive power was limited (F1 score = 0.353). **d,** Immunofluorescence images for ENO1 (top) and PDHB (bottom) reveal conserved single-cell heterogeneity across multiple cell lines. e, Immunofluorescence images of CYP2E1 reveal single-cell heterogeneity. **f-h**, Enzymes have different physical properties from CCD proteins. **f,** Heterogeneously expressed enzymes and CCD proteins have comparable RNA expression

levels. **g, h** Differences in physical properties, indicated by protein melting points **(g)** and protein disorder **(h)** between variable enzymes and cell cycle dependent proteins. Mann-Whitney U test with Benjamini-Hochberg correction. For box plots: center line, median; box, first (Q1) and third (Q3) quartiles; whiskers, 1.5× interquartile range (IQR) below Q1 and above Q3.

3. Macro-Spatial Factors of Heterogeneity: Are the localization and intensities of enzymes in neighboring cells statistically independent? Alternatively, do they depend on the cells being direct neighbors? Examining spatial dependencies could offer additional insights into the heterogeneity reported.

We thank the reviewer for this important comment. Differences in the population context could indeed result in functional phenotypic differences of human cells as demonstrated in the past[1,2]. However, the HPA dataset was acquired using confocal microscopy at 63× magnification to enable detailed subcellular localization of all human proteins. As each image contains only 5–20 cells, comprehensive analysis of microenvironmental influences on protein expression is unfortunately limited by the dataset's scale and resolution. We have generated a novel 4i multiplexed imaging dataset (Fig 4) and observed no correlation of metabolic protein expression to cell or nuclear size, but several enzymes including from the TCA cycle and oxidative phosphorylation correlate positively with the cytoplasmic-nuclear aspect ratio (Fig. 4d). In addition, our results suggest that cellular metabolism operates along a continuum of metabolic states and that different enzymes possess distinct relationships with different metabolic markers. We did not analyse the effect of the microenvironment on metabolic states at this point, and believe that more complex biological systems like organoids or tissues are better suited for this.

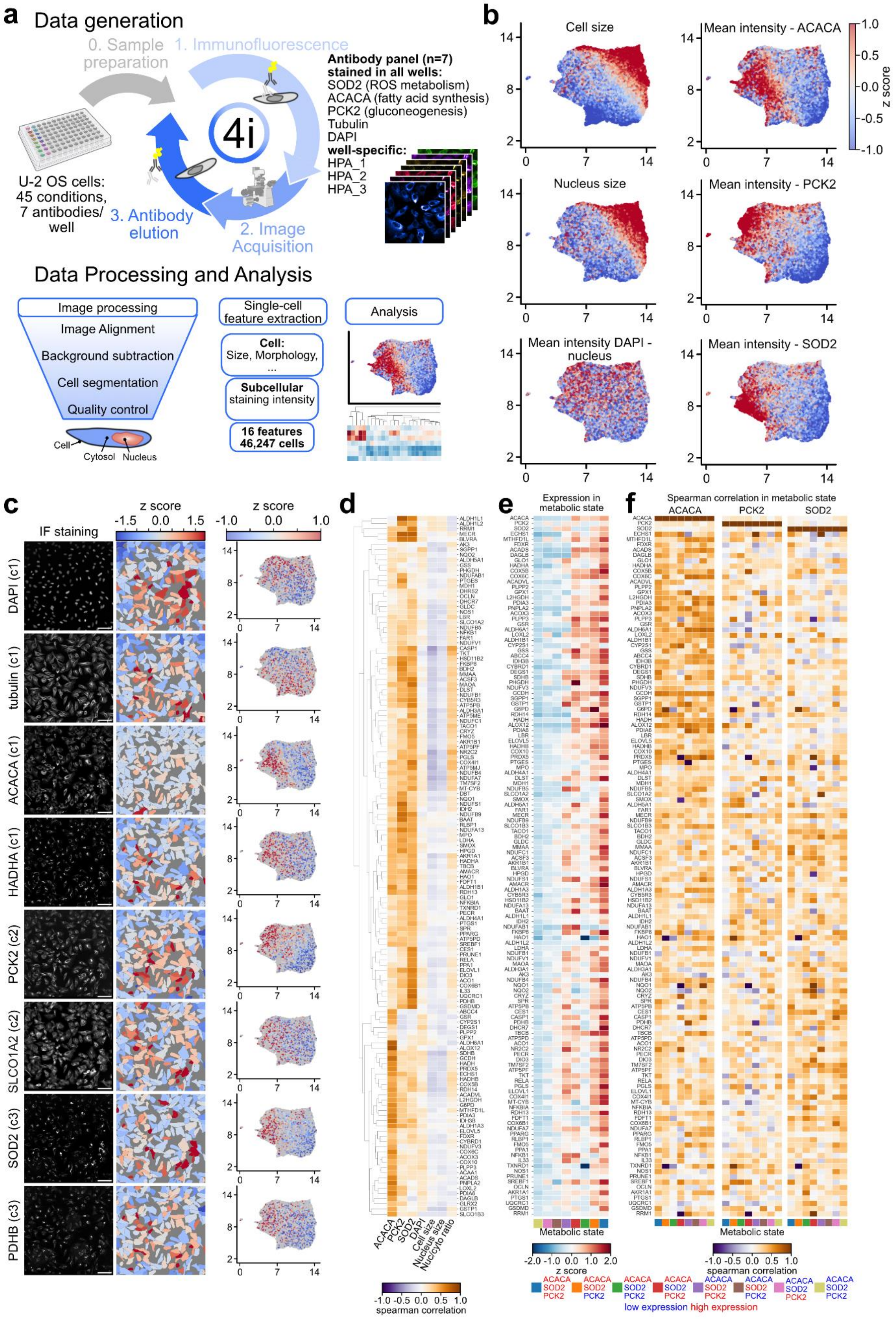

Fig. 4: Metabolic heterogeneity establishes a continuous metabolic state landscape

a, Experimental Design of a three cycle 4i experiment. Wells were stained for three rate limiting enzymes (SOD2, ACACA and PCK2), and three well-specific additional enzymes, along with DAPI, and tubulin. Cells were segmented and single-cell morphological and staining features

were extracted for analysis. **b,** cells were projected into a 2D feature landscape using UMAP dimensionality reduction on single cell features. UMAP data points colored by different features. **c,** Example data from one well; left: immunofluorescence images for 7 antibodies and DAPI from one 4i experiment (greyscale, scalebar corresponds to 30 µm); middle: mean intensity per cell visualized on the cell masks from the left panel; right: mean intensity per cell visualized on UMAP obtained in b). **d,** Heatmap visualizing spearman correlation between mean protein and metabolic marker expression. **e,** Heatmap displaying the mean expression (z score) of proteins across 8 metabolic clusters defined by high/low expression of the three metabolic markers. **f,** Heatmap displaying the spearman correlation between protein and metabolic markers (from left to right: ACACA, PCK2 and SOD2) in each metabolic cluster.

Here are some minor comments comments which can be addressed:
* Multi-Localizing Enzymes: Can the authors comment on or refer to prior work that confirms the IF detection is not an artifact of background fluorescence, particularly in dense compartments like the nucleus?

We thank the reviewer for this important comment and concern. We acknowledge that the nucleus can be a source of nonspecific antibody binding due to its charge and high density, as we described in Stadler et al[3]. However, the degree of protein multilocalization in the HPA dataset is similar to other orthogonal datasets derived using mass spectrometry based methods or high-throughput fluorescent protein tagging[4–8]. HPA antibodies are also carefully validated using orthogonal antibody validation strategies, such as Protein Arrays, an independent antibody targeting a different epitope, GFP tagging, gene silencing and IP-MS[3,9–11]. Based on the antibody validation, literature and IF staining, each protein location is given a reliability score at a three tier level: Uncertain: Validated with Protein Arrays and no contradicting localisation data in UniProt; Supported: Validated with Protein Arrays and supporting (independent) localisation data in UniProt; Enhanced: Validated with Protein Arrays, no contradicting localisation data in UniProt, and validated with two independent antibodies, GFP tagging or gene silencing. The validation procedures are clearly described at https://www.proteinatlas.org/about/antibody+validation. We also summarized the antibody validation in the methods section “HPA antibodies and validation”. The data quality of the protein localization data in the HPA has been extensively reviewed multiple times[7,12,13]. We have also added these references to the Methods section.

There is also some recent work by the group of Dr. Stephen Quake (in revision for Nature) investigating low intensity nuclear stainings in the HPA. They have noted that much of the low intensity nuclear stainings in the HPA, corresponds to transcription factors (TF’s) that are ubiquitously expressed in single cell sequencing data. In their paper they present an analysis ubiquitously expressed TF´s in the Tabula Sapiens dataset. They analyzed the HPA and identified stained tissues with 78 of the 100 TF’s in 19 tissues in common with Tabula Sapiens. The fraction of low TF’s detected in 18 or 19 tissues was 0.67. While this is in a sense irrelevant to our study, it is a nice external validation of the low intensity nuclear stainings that are often questioned in HPA, and will be published shortly.

* Fig. 2 Caption: The caption for Fig. 2 needs correction—it currently omits Fig. 2c and mislabels other panels.

We thank the reviewer for this observation and have updated the figure legend to depict the figure’s content accurately.

* Define CCD and Non-CCD: Please provide definitions for “CCD” and “non-CCD” for clarity.

We thank the reviewer for this observation. CCD was previously only defined in the methods section. We have now defined CCD as “cell cycle dependent” on page 5.

* Terminology Consistency: The terms “moonlighting” and “multifunctional” are used inconsistently, as are “variability” and “heterogeneity.”

We thank the reviewer for this observation. We chose to use “variability” or “variable” interchangeably with “heterogeneity” or heterogenous” for improved readability and flow. Moonlighting proteins are a subclass of multifunctional proteins and are defined as proteins where the same isoform performs unrelated functions[14,15]. As HPA antibodies are designed to target many isoforms of the same protein, we use the term “multifunctional” for our findings, and reserve “moonlighting” when referencing external databases.

Reviewer #2 (Remarks to the Author):

The results about metabolic enzymes are interesting, however, the images of metabolic enzymes are not from the "same single cells". Thus, the findings presented are ill-defined.

The suggestion is to use CyCIF, CODEX, IMC, MIBI, or similar method to validate the biological conclusions.

We thank the reviewer for this concern and appreciate that the data reported in this study represents findings from independent experiments using thousands of well-validated antibodies across multiple cell lines (and tissues). We do not report our findings as being derived from the “same single cells” but rather as a meta-analysis across these thousands of experiments. We report that many of the analysed proteins localize to multiple cellular compartments and display single-cell heterogeneity. Consequently, we are unsure which of our findings the reviewer believes to be “ill-defined”. Please find below a list of our main conclusions and findings, and a quick explanation of why we do not consider them “ill-defined”:

- We report the subcellular organisation of the metabolic proteome including multilocalizing enzymes. It has been extensively reviewed that imaging-based spatial proteomics using immunofluorescence is a powerful method to map the location of the proteome in single cells[16,17]. In addition, our findings regarding the large amount of multilocalizing proteins are supported by various independent studies using various methods including high-throughput gene tagging[4,5] or subcellular fractionation followed by mass spectrometry[6,8].
- The single-cell resolution in our data enables the analysis of single-cell heterogeneity in protein expression or location between individual cells. We report that a large fraction of metabolic enzymes are heterogeneously expressed. This fraction is significantly higher for enzymes than the remaining human proteome. Most enzymes vary independently of the cell cycle. Both these conclusions can be obtained using high-throughput immunofluorescence in independent experiments as shown previously[13]. As metabolic heterogeneity seems to be largely independent of cell cycle progression, we suggest that it establishes metabolic states. We agree with the reviewer that we would need data from the same single cells to define these metabolic states and determine which enzymes and pathways are correlated. However, we did not define metabolic states in the original manuscript. Instead, we state explicitly that our

> “findings offer a robust baseline for future research into genetic, environmental, and pharmacological perturbations of metabolic cellular phenotypes.“

The development of multiplexed imaging technologies, such as the ones suggested by the reviewer, has enabled the study of up to 30-100 proteins with subcellular resolution in the same single cells[18], 20x-70x less than the number of proteins covered in this study. We acknowledge that a multiplexed imaging dataset would be valuable for dissecting the origins of cellular heterogeneity for a small subset of proteins in this study. It would also allow studying the functional impact of cell-cell heterogeneity across different conditions.

While proteome-wide multiplexed immunofluorescence is not feasible using any technology available, we like the suggestions of using some kind of multiplexed imaging, and have performed a 4i multiplexed microscopy experiment to validate the existence of metabolic states in the same individual cells. Here, we stained U-2OS cells with three metabolic markers, DAPI, and tubulin. Each condition was then costained with three different, additional metabolic enzymes. This dataset results in experimental data for 135 metabolic enzymes co-stained with the same metabolic markers in 46,274 cells. We validate that metabolic states exist in a continuum under steady-state conditions.

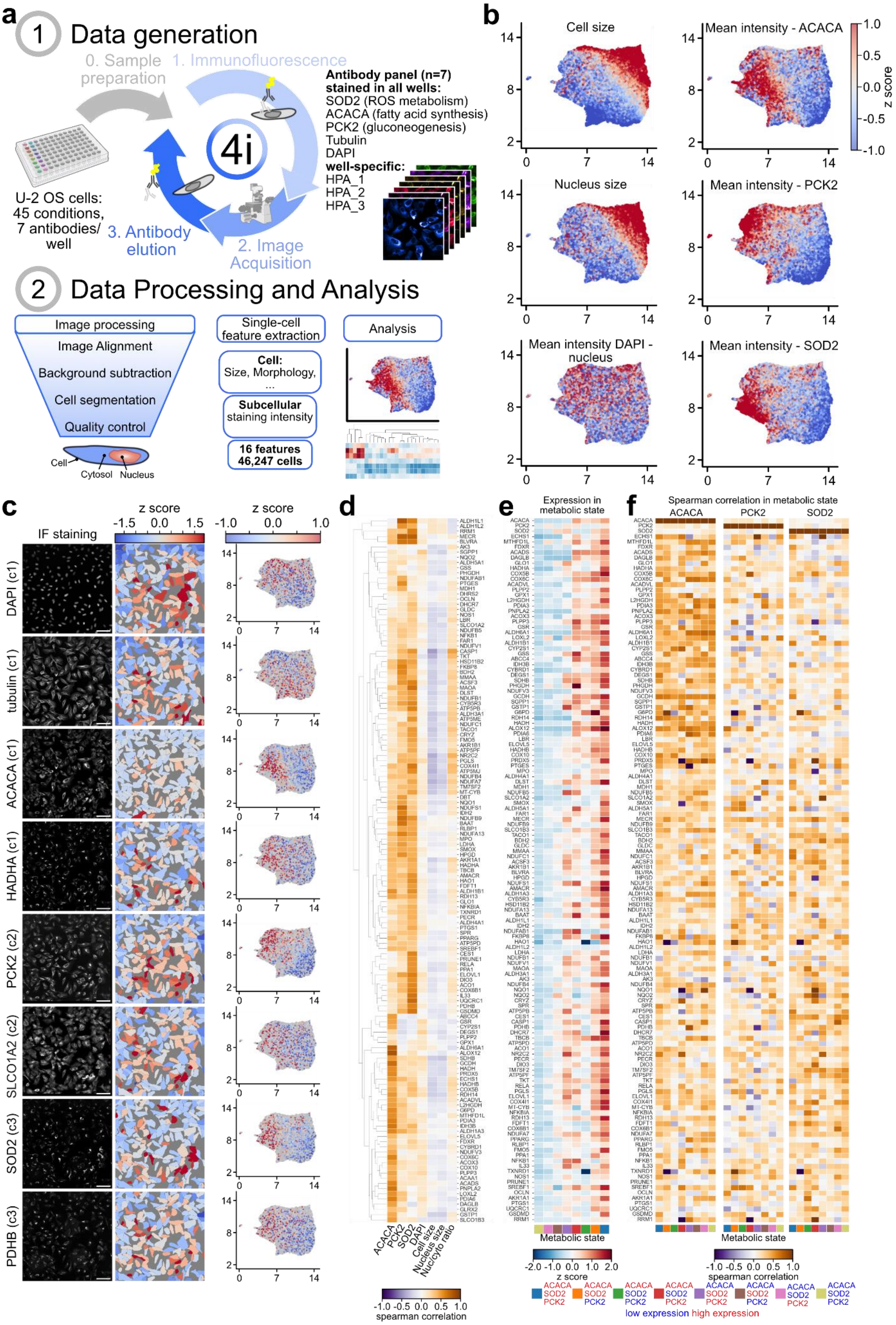

Fig. 4: Metabolic heterogeneity establishes a continuous metabolic state landscape

a, Experimental Design of a three cycle 4i experiment. Wells were stained for three rate limiting enzymes (SOD2, ACACA and PCK2), and three well-specific additional enzymes, along with DAPI, and tubulin. Cells were segmented and single-cell morphological and staining features were extracted for analysis. **b,** cells were projected into a 2D feature landscape using UMAP

dimensionality reduction on single cell features. UMAP data points colored by different features. **c,** Example data from one well; left: immunofluorescence images for 7 antibodies and DAPI from one 4i experiment (greyscale, scalebar corresponds to 30 µm); middle: mean intensity per cell visualized on the cell masks from the left panel; right: mean intensity per cell visualized on UMAP obtained in b). **d,** Heatmap visualizing spearman correlation between mean protein and metabolic marker expression. **e,** Heatmap displaying the mean expression (z score) of proteins across 8 metabolic clusters defined by high/low expression of the three metabolic markers. **f,** Heatmap displaying the spearman correlation between protein and metabolic markers (from left to right: ACACA, PCK2 and SOD2) in each metabolic cluster.

In our original manuscript, we also provided extensive validations of our findings using orthogonal technologies to reduce technological bias. For example, we compared our findings of protein expression heterogeneity to data derived from clonal expansion of genetically engineered cell lines with fluorescently-tagged protein of interest[5] and to proteomics measurements in single liver hepatocytes[19]. Similarly, the HPA protein location data has been extensively compared internally and externally to mass spectrometry-based subcellular proteomics datasets and fluorescent-tagging datasets to validate our claims about protein multilocalization[4–7,20].

Also, it would be important to remove claims about spatial proteomics - which is nowadays referring to the "same single cells" not a historical library of datasets from ancient images.

We acknowledge that the term “spatial proteomics” refers to both the study of subcellular protein localization and the spatial mapping of proteins in tissues. However, we disagree with the statement that “spatial proteomics” is limited to studying protein location in the same single cells. A recent Nature editorial[21] defines different methods for spatial proteomics with single-cell and bulk resolution. These include optical methods such as high-throughput gene tagging or immunofluorescence, and multiplexed imaging technologies. But the field also includes mass-spectrometry based methods where protein location is inferred from the study of thousands or millions of cells, for example after cellular fractionation, through proximity labelling or interactomics methods. Altogether, the field consists of many different, complementary technologies, each with its own advantages and disadvantages[16,17,22,23].

We would like to highlight that data quality does not deteriorate over time. By automating many aspects of sample preparation and microscopy within the Subcellular Section of the Human Protein Atlas, we aim to maximize reproducibility of the experiments across the years. Thorough quality control ensures a high standard of the generated and presented data, and extensive curation efforts ensure the database, annotations and reliability scores are up to date for every yearly HPA release. This ensures that the HPA is always up-to-date and prevents it from turning into a “historical” database. This study used data from HPAv23, which was released less than two years ago. Most importantly, reanalysis of this previously generated data has enabled many novel biological findings in this project and allowed for the profiling of cellular metabolism at unprecedented scale with single-cell and subcellular resolution.

Reviewer #3 (Remarks to the Author):

We are grateful to Reviewer 3 for their comprehensive review and appreciate their constructive comments and well-structured feedback. The suggestions to provide additional detail on the statistical methods used were very helpful in improving the manuscript.

This study presented results from an analysis of the metabolic proteome data in the HPA (HPAv23 Subcellular Section, 17,180 IF images). This is a comprehensive, unique resource for studying spatial biology of cellular metabolism. The study generated some interesting biological insights (e.g., localization, dynamics, multi-localization of some enzyme expressions) and verified/expanded some previous understanding on spatial metabolism. While those were exciting, insightful, the manuscript was written in a highly descriptive way. The data analysis and the methods were not described in sufficient detail to establish statistical rigor of the analysis, which I think is important when it comes to IF image data analysis and can significantly affect conclusions or accuracy in this study.

We thank the reviewer for this comment. The analysis presented in this manuscript is based on manual annotations derived directly from the Human Protein Atlas, Subcellular Section. We addressed the specific concerns regarding the method section and statistical analysis further down.

Also, the work is not validated/compared (at least partially) against orthogonal data/technologies that directly inform spatial metabolism (e.g., MALDI), leaving one wondering how precise/informative the observations/conclusions are.

We thank the reviewer for this important comment. While we have discussed data from studies using single-cell MALDI in the context of single cell heterogeneity of metabolism[24–26], we have previously not addressed the subcellular location distribution of important metabolites in detail. To validate our protein level findings, we performed an additional analysis. Many TCA cycle enzymes and metabolites are known to be present in the nucleus[27,28]. We added an analysis of protein multilocalization specifically for TCA cycle enzymes and show that almost 30% localize to both the nucleus and the cytoplasmic compartment (i.e. cytosol or mitochondria) in the HPA dataset. This validation confirms that both protein and metabolite localization can be important for cellular organisation. To our knowledge, single-cell MALDI studies reporting not only the abundance but also subcellular localization of metabolites are rare, and there is no database of metabolite subcelllular localization. Subcellular metabolomics or lipidomics is performed in bulk on organelle enriched fractions derived by cellular fractionation. Interestingly, these studies reveal that “unlike proteins, where more than half exhibit unique cellular localizations, most lipids demonstrated multiple localizations”[29]. These differences are likely due to the higher diffusion rate across membranes by metabolites and lipids compared to proteins. We have included these insights in the conclusion.

Comments:
Generally, when a concept is defined, it is necessary to quantify it with a mathematical/statistical metric, and describe what kind of test (and why that) was used to determine the value. For example:

We thank the reviewer for these comments and fully agree with this statement. We aim to address them below.

Page 4. “metabolic enzymes across all pathway groups exhibit a significantly higher degree of heterogeneous expression ”. It is unclear how this heterogeneity (related to dynamics) is quantified and compared against the background. What was the metric used, and what was the test used to derive the conclusion (heterogeneous vis non-heterogeneous). Missing methodological descriptions.

The sentence refers to interpretation of Fig 2b quantifying the percentage of variably expressed enzymes across different metabolic pathways.

Heterogeneity in the context of this study is a categorical variable as the images generated within the Human Protein Atlas database are manually annotated for protein localization and heterogeneity following a strict standard operating procedure (see methods). To address the reviewer's concerns and better reflect the nature of our data, we have updated the sentence highlighted by the reviewer:

"Strikingly, an even larger percentage of metabolic enzymes across all pathway groups display heterogeneous expression (805 of 2,126 enzymes, 37.9%, i.e., 68.4% higher than whole proteome, p=4.7e-47, binomial test; Fig. 2b)."

We have also extended the method section "Identification of proteins with cell-to-cell heterogeneity as well as multilocalizing proteins" to explicitly state that heterogeneity and multilocalization in the context of this study are categorical variables. We also added additional references, where the data generation and annotation in the HPA has been extensively reviewed.

Finally, we added an additional validation analysis to address the reviewer's concern about a statistical quantification for single-cell heterogeneity. We extracted pixel intensities from individual U2OS cells (nucleus, cytosol, and whole cell) in the Human Protein Atlas (HPA) dataset and computed the median absolute deviation (MAD) per image. As anticipated, images manually annotated as "scv" exhibited significantly higher MAD values than those with stable expression.
However, the limited number of cells per image (5-20) in the HPA dataset constrains the statistical robustness of this approach. Therefore, we relied on the original manual annotations for identifying single-cell variability and present this analysis as complementary validation in Extended Data Fig. 2a.

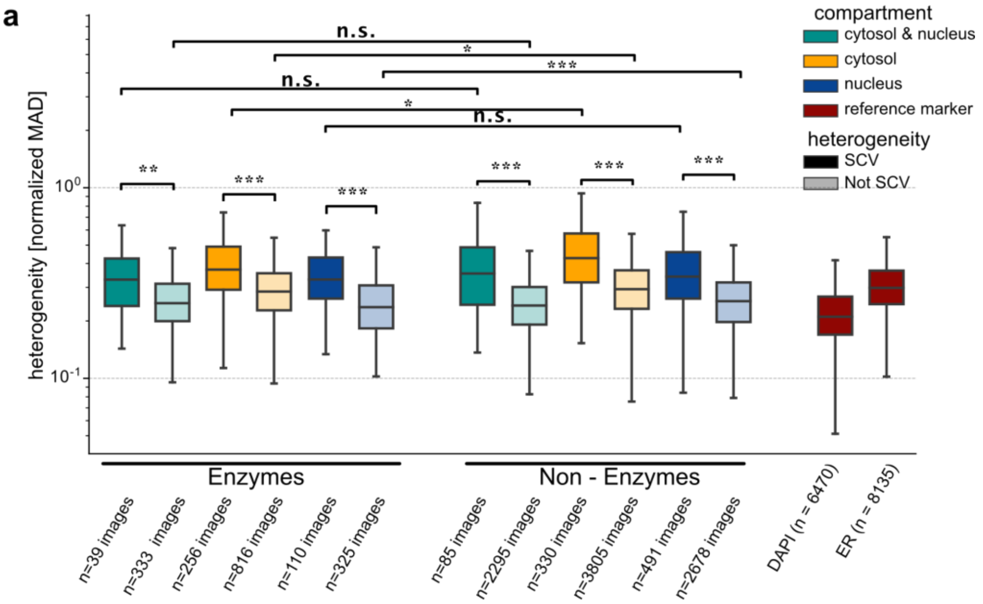

Extended Data Fig. 2: The variable metabolic proteome in the HPA Subcellular Section
a, The normalized median absolute deviation was calculated between individual cells in the same image as a measure of protein expression variability for different groups of proteins (enzymes and proteins in different cellular compartment that are variably expressed or not based on manual annotations from HPAv23.

Page 4, “we performed a gene ontology (GO)-term enrichment analysis of variably and stably expressed enzymes against the background of the entire metabolic proteome”. How were variably and stably expressed enzymes defined from the data? What were the quantification metric and cutoff to classify them?

The sentence refers to interpretation of Fig 2d. As addressed above, heterogeneity is a categorical variable derived directly from manual annotations in the Subcellular Section of the Human Protein Atlas (see methods).

Page 6, “more than half of all metabolic proteins are multi localizing ”. Only a brief description of “multi-localizing” was found in Methods with no detailed description on quantification. This is insufficient and likely inaccurate, as the imaging data can have various type/level of noise, which can significantly affect the conclusions or textual description.

The reviewer raises important concerns on different noise levels across the experiments. These are minimised through automation of the sample preparation workflow and the multi-step annotation procedure in the database ensuring high quality of the data. The HPA data has been extensively reviewed before and a detailed overview about the annotation and quality control is provided here[7,12,13]. We added these references to the Materials and Methods section. In short, immunofluorescence stainings are performed in 96 well format. The noise level for each 96 well plate is assessed through a negative control. Cellular localisations with staining intensities similar to the negative control are annotated as negative. Each protein location is assigned a reliability score based on the antibody validation, available literature and the immunofluorescence staining at a three tier level: Uncertain: Validated with Protein Arrays and no contradicting localisation data in UniProt; Supported: Validated with Protein Arrays and supporting (independent) localisation data in UniProt; Enhanced: Validated with Protein Arrays, no contradicting localisation data in UniProt, and validated with two independent antibodies, GFP tagging or gene silencing. The validation procedures are clearly described at https://www.proteinatlas.org/about/antibody+validation and we summarized it in the methods section “HPA antibodies and validation”.
Furthermore, the localisation data reported in the Human Protein Atlas has a high overlap with localisation data derived from orthogonal methods, for example mass spectrometry based subcellular proteomics or high throughput gene tagging, which has previously been reported in our own and in independent work[4,5,7,20]. In addition, many complementary studies report similar levels of multilocalizing proteins compared to the HPA[4,6,8].

Therefore, we are confident in the amount of multilocalizing proteins reported in the HPA and the confidence level for each protein location is reported publicly available at proteinatlas.org.

Some important methodological details are missing:
Page 3, “To quantify the similarity of subcellular distribution for different sets of metabolic enzymes, we embedded the spatial information from the images into a compressed representation using a machine learning approach (Methods).30” I could not find the corresponding description in Methods. Reference [30] did not describe how to embed spatial information with the protein expressions onto high dimensional or UMAP representations shown in Fig. 1d and 1g.

The reviewer raises an important question on data presentation and method description. The reference provided (Ouyang *et al.,* Nature Methods 2019) describes a deep learning model that accurately predicts protein localization in immunofluorescence data[30]. This

model was trained on the entire HPA image dataset. The protein staining features extracted by the model can be represented in 2D UMAP space and reveal a separation of images by protein localization. In this manuscript, we extracted the coordinates from the UMAP generated by Ouyang et al and visualized different groups of proteins (e.g., enzymes in cholesterol metabolism, Fig 2g). We calculated the average pairwise cosine distance for enzymes in the same pathways to identify compartmentalized metabolic pathways. The original reference describes the deep learning model used for feature extraction in great depth and provides information about how to access the metadata and UMAP coordinates: *"A web application was developed and deployed with the ImJoy platform, where users can interact with the UMAP and the images with associated metadata (https://tinyurl.com/y6nhf5bo). This provides a new way to explore large online resources, such as the HPA database."*

We agree that the highlighted sentence might be misinterpreted, as we did not apply the machine learning model described in Ouyang et al ourselves, but instead used the direct output of their study for our analysis. We have updated this section in the main text and methods to clarify how the data for the analysis was obtained.

Methods: "Ouyang et al embedded the spatial information from the entire HPA image dataset into a compressed representation using a machine learning approach. We obtained these embeddings and visualized them as a 2D projection of a UMAP (Extended Data Fig. 1e)."
Main text: "To quantify the similarity of subcellular distribution for different sets of metabolic enzymes, we extracted a compressed representation of the spatial information in the entire HPA image dataset from Ouyang et al."

In addition, we have implemented an interactive UMAP plugin on the HPA website (https://www.proteinatlas.org/humanproteome/subcellular/location+umap). This plugin enables users to browse the entire HPA dataset and explore patterns of protein localization of individual proteins, across different metabolic pathways or in different subcellular localizations. We added this link in the main text and the method section.

Minor comments:
Page 3: "Of these genes, we have measured the subcellular distribution for 2,126 proteins". How many genes these 2,126 proteins correspond to?

We thank the reviewer for this comment. The HPA is a gene-centric protein database. 2,126 proteins refer to an equal number of genes. We added a short sentence in the main text for clarification.

Fig. 1g, in the color legend, the colored dots and the text labels are unaligned.

We thank the reviewer for their attention. We updated the figure and aligned legend and text.

Fig. 2b, the Y axis label "scv" needs to be spelled out.

We thank the reviewer for their attention. We updated the figure axis.

Fig.2e, "HPA" appeared twice in the column labels.

We thank the reviewer for their attention. "HPA" refers to the same antibody stained in multiple cell lines. We updated the figure design to improve its clarity.

Fig. 2f, “mNG” appeared twice in the column labels.

We thank the reviewer for their attention. In the left column the mNG signal is visualized in grey scale, while in the middle column mNG is visualized using a high contrast colormap to improve the contrast between individual cells. We have updated the column labels.

Fig. 4 is largely illegible, showing largely just the variability. I think it needs to be presented in some other ways.

We thank the reviewer for their attention. Our goal was to provide a visualization of how protein expression heterogeneity can impact the entire subcellular metabolism network. To improve readability, we removed Figure 4 and highlighted individual pathways instead (Extended Data Fig. 3 and Extended Data Fig 5). These were obtained from the Metabolic Atlas[31] and customized using HPA information. We generated these pathway maps allowing to explore protein localization and expression heterogeneity for all Human1 pathways. These can be explored in the newly established Interactive pathway explorer at https://www.proteinatlas.org/humanproteome/subcellular/metabolic+proteome.

References

1. Kramer, B. A., Sarabia Del Castillo, J. & Pelkmans, L. Multimodal perception links cellular state to decision-making in single cells. *Science* **377**, 642–648 (2022).
2. Metz-Zumaran, C. *et al.* The population context is a driver of the heterogeneous response of epithelial cells to interferons. *Mol. Syst. Biol.* **20**, 242–275 (2024).
3. Stadler, C. *et al.* Systematic validation of antibody binding and protein subcellular localization using siRNA and confocal microscopy. *J. Proteomics* **75**, 2236–2251 (2012).
4. Cho, N. H. *et al.* OpenCell: proteome-scale endogenous tagging enables the cartography of human cellular organization. *Science* **375**, eabi6983 (2022).
5. Reicher, A. *et al.* Pooled multicolour tagging for visualizing subcellular protein dynamics. *Nat. Cell Biol.* **26**, 745–756 (2024).
6. Geladaki, A. *et al.* Combining LOPIT with differential ultracentrifugation for high-resolution spatial proteomics. *Nat. Commun.* **10**, 331 (2019).
7. Thul, P. J. *et al.* A subcellular map of the human proteome. *Science* **356**, (2017).
8. Itzhak, D. N., Tyanova, S., Cox, J. & Borner, G. H. Global, quantitative and dynamic mapping of protein subcellular localization. *eLife* **5**, e16950 (2016).
9. Nilsson, P. *et al.* Towards a human proteome atlas: high-throughput generation of mono-specific antibodies for tissue profiling. *Proteomics* **5**, 4327–4337 (2005).
10. Stadler, C. *et al.* Immunofluorescence and fluorescent-protein tagging show high correlation for protein localization in mammalian cells. *Nat. Methods* **10**, 315–323 (2013).
11. Edfors, F. *et al.* Enhanced validation of antibodies for research applications. *Nat. Commun.* **9**, 4130 (2018).
12. Barbe, L. *et al.* Toward a confocal subcellular atlas of the human proteome. *Mol. Cell. Proteomics MCP* **7**, 499–508 (2008).
13. Mahdessian, D. *et al.* Spatiotemporal dissection of the cell cycle with single-cell proteogenomics. *Nature* **590**, 649–654 (2021).
14. Chen, C., Zabad, S., Liu, H., Wang, W. & Jeffery, C. MoonProt 2.0: an expansion and update of the moonlighting proteins database. *Nucleic Acids Res.* **46**, D640–D644 (2018).
15. Chapple, C. E. *et al.* Extreme multifunctional proteins identified from a human protein interaction network. *Nat. Commun.* **6**, 7412 (2015).
16. Strack, R. Subcellular spatial proteomics. *Nat. Methods* **21**, 2227–2227 (2024).
17. Christopher, J. A. *et al.* Subcellular proteomics. *Nat. Rev. Methods Primer* **1**, 32 (2021).
18. Hickey, J. W. *et al.* Spatial mapping of protein composition and tissue organization: a primer for multiplexed antibody-based imaging. *Nat. Methods* **19**, 284–295 (2022).
19. Rosenberger, F. A. *et al.* Spatial single-cell mass spectrometry defines zonation of the hepatocyte proteome. *Nat. Methods* **20**, 1530–1536 (2023).
20. Reicher, A., Koren, A. & Kubicek, S. Pooled protein tagging, cellular imaging, and in situ sequencing for monitoring drug action in real time. *Genome Res.* **30**, 1846–1855 (2020).
21. Remmel, A. Where do proteins go in cells? Next-generation methods map the molecules' hidden lives. *Nature* **640**, 556–560 (2025).
22. Breckels, L. M. *et al.* Advances in spatial proteomics: Mapping proteome architecture from protein complexes to subcellular localizations. *Cell Chem. Biol.* **31**, 1665–1687 (2024).
23. Lundberg, E. & Borner, G. H. H. Spatial proteomics: a powerful discovery tool for cell biology. *Nat. Rev. Mol. Cell Biol.* **20**, 285–302 (2019).
24. Bien, T., Koerfer, K., Schwenzfeier, J., Dreisewerd, K. & Soltwisch, J. Mass spectrometry imaging to explore molecular heterogeneity in cell culture. *Proc. Natl. Acad. Sci.* **119**, e2114365119 (2022).
25. Rappez, L. *et al.* SpaceM reveals metabolic states of single cells. *Nat. Methods* **18**, 799–805 (2021).
26. Capolupo, L. *et al.* Sphingolipids control dermal fibroblast heterogeneity. *Science* **376**, eabh1623 (2022).
27. Kafkia, E. *et al.* Operation of a TCA cycle subnetwork in the mammalian nucleus. *Sci.*

Adv. **8**, eabq5206 (2022).
28. Li, W. *et al.* Nuclear localization of mitochondrial TCA cycle enzymes modulates pluripotency via histone acetylation. *Nat. Commun.* **13**, 7414 (2022).
29. Haas, D. T. *et al.* C-COMPASS: A Neural Network Tool for Multi-Omic Classification of Cell Compartments. 2024.08.05.606647 Preprint at https://doi.org/10.1101/2024.08.05.606647 (2024).
30. Ouyang, W. *et al.* Analysis of the Human Protein Atlas Image Classification competition. *Nat. Methods* **16**, 1254–1261 (2019).
31. Robinson, J. L. *et al.* An atlas of human metabolism. *Sci. Signal.* **13**, eaaz1482 (2020).
